# Nuclear Quantum Confinement Enables Robust Deuterium Bonds for Highly Reversible Aluminum Anodes

**DOI:** 10.1002/anie.202508422

**Published:** 2025-07-09

**Authors:** Hao Cheng, Yao Lu, Zheng Li, Zibo Chen, Chao Chen, Xinyi Li, Hailin Yu, Adham Hashibon, Zhongliang Tian, Guanjie He

**Affiliations:** ^1^ School of Metallurgy and Environment Central South University Changsha 410083 China; ^2^ National Engineering Research Centre of Low‐carbon Nonferrous Metallurgy Changsha 410083 China; ^3^ National Energy Metal Resources and New Materials Key Laboratory Changsha 410083 China; ^4^ School of Materials Science and Engineering Hunan University of Science and Technology Xiangtan 411201 China; ^5^ Institute for Materials Discovery University College London 107 Roberts Building London WC1E 7JE UK; ^6^ Department of Chemistry University College London 20 Gordon Street London WC1H 0AJ UK

**Keywords:** Aluminum anode, Aqueous aluminum battery, Hydrogen bond, Nuclear quantum confinement, Solvation sheath

## Abstract

The hydrogen evolution reaction (HER) fundamentally limits aluminum electroreduction in aqueous electrolytes by dominating interfacial charge transfer. Here, we suppress HER by engineering deuterium bonds (D‐bonds) through nuclear quantum effects, confining D between D₂O and DMF molecules. This quantum confinement weakens hydrogen delocalization and restructures the Al^3+^ solvation sheath, reducing water activity kinetically and thermodynamically. The regulated electrolyte enables uniform aluminum nucleation and dense plating layers, achieving 569 h (0.05 mA cm^−2^) and 379 h (0.1 mA cm^−2^) cycling stability in the 2D_2_O/1DMF electrolyte, which outperforms traditional sulfate electrolytes by 3.6 and 6.1 times, respectively. Our work uniquely leverages nuclear quantum confinement to engineer robust D‐bonds, simultaneously suppressing HER and enabling atomic‐level control over aluminum ion solvation structures for unprecedented Al redox reversibility in sulfate electrolytes. This exemplification pushes the electrolyte engineering from extensive component adjustment to quantum precision engineering, which provides an innovative solution for the high‐activity water‐based battery system

## Graphical abstract

Quantum confinement by leveraging D as a quantum bridge is engineered to tailor stronger intra‐ and intermolecular D‐bonds, effectively suppressing water reactivity and achieving exceptional HER inhibition. The induced Al^3+^ solvation structures further inhibit the decomposition of water at the interface and improve Al redox reversibility.

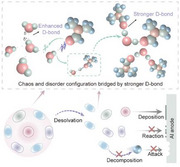

## Introduction

The electrochemical stability window of water poses a fundamental constraint on the electroreduction of Al^3^⁺ in aqueous solution, consequently necessitating the use of molten salts or nonaqueous solution to achieve high reversibility in aluminum electrochemistry.^[^ ^1^ ^]^ The electron‐consuming hydrogen evolution reaction (HER) preferentially occurs at a low applied potential, thereby hindering Al^3+^ from gaining electrons (Figure S1). While triflate‐based electrolytes (Al(OTf)_3_) dominate aqueous aluminum research through weakly coordinated anions enabling high solubility and suppressed water decomposition, their inherent corrosion susceptibility drives exploration of sulfate alternatives.^[^ ^2^, ^3^ ^]^ The kosmotropic nature of SO_4_^2−^ induces strong water coordination. However, it preserves bulk hydrogen bond (HB) networks, showing minimal impact on the water stability.^[^ ^4^ ^]^ Accordingly, persistent HER in Al_2_(SO_4_)_3_ electrolytes is recognized as the principal bottleneck hindering practical aluminum anode, which has also paradoxically evaded comprehensive mechanistic investigation.

To get to the bottom of the HER, the energetic hydrogen atom transfer (HAT) undoubtedly tops the list of the reasons.^[^ ^5^ ^]^ As is well known, the minimal mass of hydrogen nuclei results in pronounced nuclear quantum effects (NQEs) for hydrogen atoms.^[^ ^6^, ^7^ ^]^ Quantum tunneling and zero‐point motion (ZPM) of hydrogen atoms, two pivotal characteristics of NQEs, facilitate HAT (Figure S2). And the dynamic and continuous HAT endows higher activity of water to release the hydrogen. In this process, a switch from the stabler covalent bond to an ordinary HB happens due to hydrogen (H) tunneling between adjacent water molecules.^[^ ^8^ ^]^ Meanwhile, the anharmonic ZPM of H makes it deviate from the position with low potential energy, resulting in the alterations of the HB length.^[^ ^9^ ^]^ Clearly, the intermolecular and intramolecular HBs serve as bridges for HAT according to the Grotthuss hopping mechanism. Hence, preventing HAT through HB control presents a potent and effective approach to suppressing HER.

Long‐range ordered tetrahedral HB networks in water promote HAT via Grotthuss hopping.^[^ ^10^ ^]^ The disruption, reconstruction, or reinforcement of this organized structure by solutes or additives can inhibit HAT by modulating the transmission pathways.^[^ ^11^, ^12^, ^13^ ^]^ Nevertheless, directional migration of solvated ions and desolvation behavior generate a water‐rich inner Helmholtz plane with continuous HB networks.^[^ ^14^ ^]^ A more negative charge density of the electrode surface facilitates the dissociation of interfacial water. Under this rule, the organic additives with polar groups could adsorb on the electrode surface to break the interfacial HB networks connectivity and isolate hydrogen sources.^[^ ^3^, ^15^ ^]^ To sum up, all current means manifest intermolecular HB manipulation erect barriers for quantum tunneling of H to stop its transfer in both bulk and interfacial regions. The restriction lessens the water dynamic reactivity to ameliorate the thermodynamic stability of the electrolyte. However, this external effect would be offset by strong polarization of Al^3+^.^[^ ^16^ ^]^ Crucially, the key to HER is the dissociation of intramolecular HBs in H_2_O, which carries out little modifications in the previously mentioned tactics. Thence, solely intermolecular HBs engineering, external restriction for H, proves insufficient to fully mitigate HER for Al anode.

The reaction behavior of H is fundamentally governed by its NQEs. A transient intramolecular HB shrinkage of approximately 0.04 Å occurs within 80 fs based on the ZPM, followed by an expansion within roughly 1 ps.^[^ ^17^ ^]^ The instantaneous relaxation of intramolecular HBs induces a dynamic pulling‐pushing to adjacent H_2_O or Al^3+^. Consequently, reducing vibrational frequency by suppressing the ZPM of H emerges as an effective strategy for modulating water activity and stabilizing Al^3+^ behavior. The macroscopic embodiment of NQEs of H is most evident in the isotope effect. Deuterium (D), with an extra neutron, possesses twice the mass of H, resulting in lower vibrational frequency and energy. This mass difference leads to the formation of stronger and shorter HBs in heavy water (D_2_O) compared to H_2_O.^[^ ^18^ ^]^ Studies have demonstrated that D_2_O exhibits approximately 9% greater HBs abundance and 10% higher HB strength than H_2_O^6^. These enhanced HBs characteristics contribute to the superior thermodynamic stability of D_2_O‐systems.^[^ ^19^, ^20^ ^]^ The attenuated pulling‐pushing effect in D_2_O systems would suppress the HER from water splitting and reduces Al^3+^ polarization. Thus, the strategic confinement of HAT, through both intrinsic (NQEs) and extrinsic (additives) mechanisms, represents a critical approach for synchronously dropping water activity and promoting efficient Al^3+^ plating.

Herein, we propose a strategy of reinforcing both intramolecular and intermolecular HBs via attenuating the NQEs of H to inhibit the HER and enhance the redox reaction of Al in Al_2_(SO_4_)_3_ electrolyte. For proof of idea, D_2_O is adopted to replace H_2_O as the solvent for Al^3+^ with a higher charge density of 364 C mm^−3^. The enhanced intramolecular HBs are evidenced by the weaker polarization caused by strong electrostatic action. We further introduce N, N‐dimethylformamide (DMF) as a cosolvent, utilizing its high Gutmann donor number (26.6 kcal mol⁻¹) to restrict free D_2_O through confined intermolecular HBs.^[^ ^21^ ^]^ Significantly stable water molecules, HB strength, and the solvation structure via internal and external synergy are demonstrated through theoretical computation and experimental characterization. To substantiate the effectiveness of the strategy, Al||Al symmetric cell with engineered electrolyte achieves an exceptional cycling stability of 569 h at 0.05 mA cm^−2^/0.05 mAh cm^−2^ and 379 h at 0.1 mA cm^−2^/0.1 mAh cm^−2^. Notably, Al||Ti asymmetric cell delivers an impressive coulombic efficiency of 95.56% (100 cycles) at 0.05 mA cm^−2^. This work deciphers the mechanistic interplay between hydrogen quantum behavior and HER dynamics, establishing HBs network engineering as a universal exemplification for precise water activity regulation in Al_2_(SO_4_)_3_ solution for aluminum electrochemistry.

## Results and Discussion

### Engineering Stronger HBs by Harnessing NQEs

**Figure 1:**
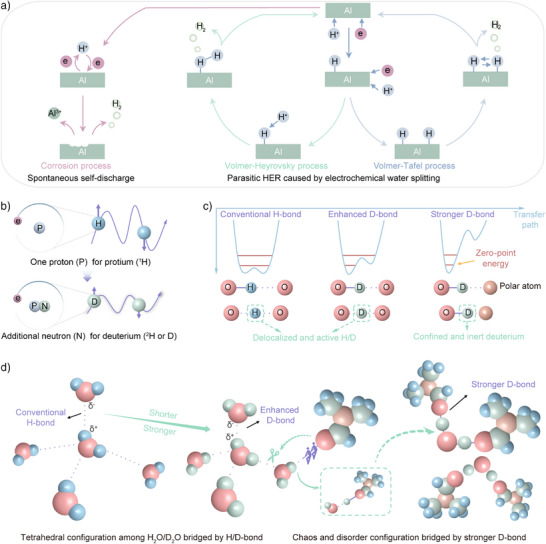
Schematic illustrations for the HER and HBs regulation. a) Three distinct HER pathways for Al in aqueous systems. b) Atomic structures comparison between H and D. c) Classification and characteristics of HBs. d) Transformation of HB network configurations through suppression of NQEs.

In acidic aqueous systems, Al facilitates HER through three distinct mechanisms (Figure 1a). Spontaneous self‐discharge occurs via proton attack, generating hydrogen continuously. The parasitic HER proceeds through electrochemical water splitting via Volmer‐Heyrovsky and Volmer‐Tafel pathways, requiring electron transfer.^[^ ^1^ ^]^ Both intramolecular and intermolecular HBs mediate HAT during these processes. Proton relay transfer occurs along the ordered tetrahedral HBs network, exhibiting pronounced quantum behavior through continuous HBs formation and dissociation. Here, we substituted H with D, which contains an additional neutron. This 100% increase in atomic mass reduces HB vibrational frequency, inducing an obvious kinetic isotope effect (KIE) (Figure 1b).

As illustrated in Figure 1c, the contraction and relaxation of intramolecular HBs increase the positional uncertainty of H. And the attraction of adjacent water molecules lowers the HAT energy barrier to accelerate the tunneling process. These combined effects promote hydrogen atom delocalization, facilitating rapid participation in the HER. In contrast, the deuterium‐oxygen (D─O) bond exhibits greater stability than the hydrogen‐oxygen (H─O) bond due to suppressed ZPM, evidenced by a ∼3% shorter bond length.^[^ ^22^ ^]^ This enhanced bonding results in slower HER kinetics for D_2_O. However, these intrinsic differences can be attenuated by external factors, including cation effects and applied potentials. The tunneling process induces deuterium delocalization, which alone proves insufficient for effective HER suppression in complex aqueous systems. To address this limitation, we introduce polar atoms to capture D and elevate the HAT energy barrier between D_2_O and adjacent molecules. Organic compounds with high Gutmann donor numbers (DN) are particularly effective, as they form strong intermolecular HBs with water molecules. This capping effect confines deuterium within a limited spatial domain, significantly reducing its reactivity. It is well known that DMF with stronger electron‐donating ability could serve as the HB acceptor to interact with water molecule. More importantly, DMF features a cation‐philic and strongly anion‐phobic property. Thence, DMF is employed as a structural regulator of intermolecular HBs, effectively disrupting the continuous tetrahedral HB networks (Figure 1d). Leveraging the saturation and directionality of HBs, each D_2_O molecule can be coordinated by four DMF molecules, creating a disordered network reinforced by stronger D‐bonds. This restructured HB configuration demonstrates superior HER suppression capabilities.

### Theoretical Insights into Hydrogen Bond Behavior

**Figure 2:**
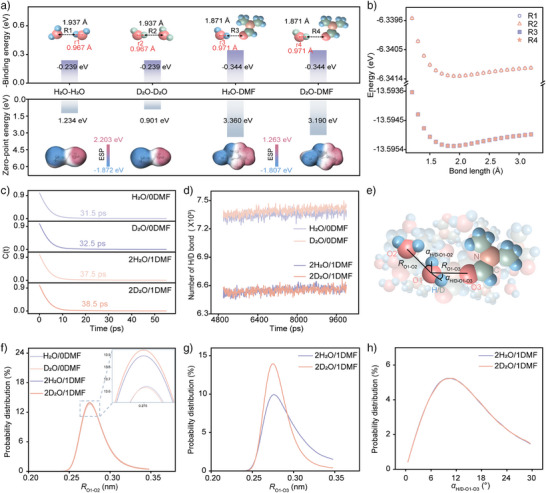
Theoretical analysis of HBs. a) Binding energy and ESP differences for various molecule pairs. b) Rigid potential energy scans of intermolecular HBs for different molecular pairs. c,d) The HBs lifespan (c) and HBs number (d) among H_2_O or D_2_O in different solution. e) Schematic representation of HBs characteristics. f–h) Probability distributions of *R*_O1‐O2_ (f), *R*_O1‐O3_ (g), and *α*_H/D‐O1‐O3_ (h) in (e).

We prepared solution systems by mixing H_2_O or D_2_O with DMF in a 1:1 volume ratio, designated as 1H_2_O/1DMF and 1D_2_O/1DMF, respectively, with DMF‐free systems (H_2_O/0DMF and D_2_O/0DMF) as controls. Frontier orbital analysis revealed stronger electrophilicity and stability of DMF (Figure S3). HBs characteristics were systematically investigated through density functional theory (DFT) and molecular dynamics (MD) simulations (Figure S4). Binding energy calculations showed stronger interactions between DMF‐D₂O (‐0.344 eV) than that of D_2_O‐D_2_O (‐0.239 eV), with a similar trend observed in H_2_O‐containing systems (Figure 2a). The 0.066 Å reduction in intermolecular distance confirmed the strong capture capability of DMF for both H_2_O and D_2_O. Accordingly, an extension of intramolecular distance of O‐H/O‐D further demonstrates the stronger competitive interaction caused by DMF. Rigid potential energy scans revealed a stabilizing effect of DMF, as evidenced by a reduction in energy (Figure 2b). Despite identical electronic properties between H and D due to their equal electron numbers, D_2_O exhibited lower zero‐point energy (ZPE) than H₂O.^[^ ^23^ ^]^ This ZPE reduction persisted in D_2_O‐D_2_O and DMF‐D_2_O compared to their H₂O counterparts. While ZPE differences between isotopes decrease in transition states due to reduced bond force constants during bond breaking, the Heisenberg uncertainty principle dictates that lower ZPE require greater activation energy. And the elevated ZPE in DMF‐containing systems originates from C─H bonds in DMF. Moreover, electrostatic potential (ESP) analysis proved DMF could balance intermolecular charge distribution, reducing reactivity.

In aqueous solutions, molecules exhibit high dynamism and cooperativity, with intermolecular HBs continuously undergoing reconstruction. D‐bonds between D_2_O molecules demonstrate an extended lifespan of 38.5 ps (Figure 2c), further increasing to 49.8 ps due to D_2_O‐DMF interactions (Figure S5a). These strengthened interactions disrupt the continuity of water networks, resulting in a reduced number of HBs among H_2_O or D_2_O molecules in DMF‐containing systems (Figures 2d and S5b). Detailed analyses of HB characteristics, including bond lengths and angles, were conducted through probability distribution statistics (Figure 2e). In DMF‐containing systems, shorter intermolecular distances and smaller angles between D_2_O molecules predominated (Figures 2f and S6). Figure 2g indicates closer proximity between D_2_O and DMF molecules, while minimal angular variations in Figure 2h. Theoretically, the *α*_H/D‐O1‐O2_ or *α*_H/D‐O1‐O3_ should approach 0° to align with HB directionality, as this configuration minimizes repulsion between polar oxygen atoms and maximizes stability.^[^ ^24^ ^]^ The observed changes in intermolecular distances and angles reflect the attenuated dragging effect caused by suppressed ZPM. These findings reveal that DMF not only captures H_2_O or D_2_O molecules but also enhances intermolecular interactions among water molecules.

### Assessment and Characterization of Electrolytes

Guided by the theoretical insights, we systematically investigated the microenvironments of H_2_O and D_2_O using advanced spectroscopic techniques. The ZPM of H induces elongation of the intramolecular O─H bond, promoting H delocalization between molecular pairs connected by HBs. This delocalized hydrogen, characterized by high reactivity, drives HBs vibrational dynamics. Fourier transform infrared (FTIR) spectroscopy revealed a broad peak at approximately 3300 cm^−1^ for pure water, corresponding to O‐H stretching vibrations (Figure S7a). In contrast, the O‐D stretching vibration shifted to 2440 cm^−1^ due to reduced vibrational frequency, with a consistent trend observed in bending vibration modes.^[^ ^25^ ^]^ The stretching vibration spectrum exhibited three distinct combination modes (Figure S7b), classified as weak, medium, and strong HBs based on wavenumber.^[^ ^26^ ^]^ Weak HBs suggest non‐coordinated or free water molecules. Medium HBs represent dimers and trimers forming partial HBs networks. And strong HBs indicate complete tetrahedral HB networks.^[^ ^14^, ^27^ ^]^

**Figure 3:**
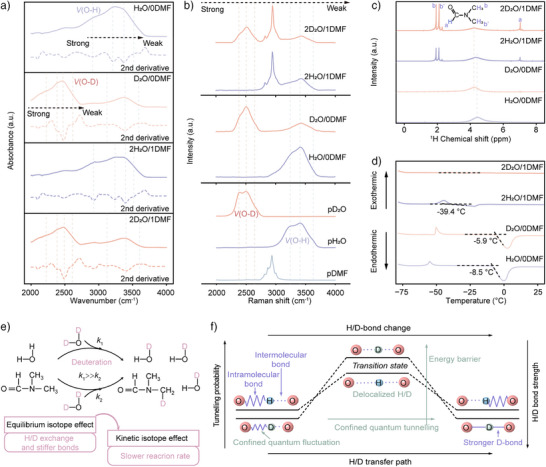
HBs characterization. a) FTIR absorbance spectra and corresponding second‐derivative analysis. b) Raman spectra. c) ^1^H NMR spectra. d) Differential scanning calorimeter (DSC) data. e) Schematic illustration of H/D exchange dynamics and its functional impact. f) Mechanism of suppressed NQEs through deuteration and molecular interactions.

Second‐derivative spectra provided enhanced resolution of HB variations, clearly reflecting changes in molecular dipole moments. The addition of DMF induces a slight blue shift in all spectral peaks (Figures 3a and S7c), implying indicating reconstruction of the HBs network and strengthening of O─H bonds. Furthermore, the areas of the OH/OD bending vibration peaks were also compared and analyzed (Figure S8), providing additional evidence for the alteration of the HBs environment. To complement these findings, Raman spectra were conducted to further analyze HBs modifications. The spectra reveal distinct symmetric and asymmetric O─H/O─D stretching modes, along with free O─H/O─D vibrations, ordered by increasing Raman shift. As shown in Figure 3b, pure heavy water (pD_2_O) exhibits greater structural organization than pure light water (pH_2_O), evidenced by a more pronounced symmetric stretching peak at 2389 cm⁻^1^ compared to 3240 cm⁻^1^ in pH_2_O.^[^ ^28^, ^29^ ^]^ The introduction of Al^3+^ and DMF diminishes this structural symmetry (Figures 3b and S9). Notably, O─H stretching vibrations appear in D_2_O‐containing systems (Figure 3a and b), primarily due to deuteration between D_2_O and DMF or residual H_2_O from hydrated aluminum salts, resulting in the formation of deuterated water and DMF species.

^1^H nuclear magnetic resonance (NMR) spectroscopy revealed significant microenvironmental changes. Consistent with the ESP results in Figure 2a, the downfield shift in H resonance frequency confirmed lessened electron density induced by DMF (Figures 3c and S10).^[^ ^14^ ^]^ This de‐shielding effect attenuates the polarity of H_2_O or D_2_O by decreasing electron cloud density, thereby reducing their reactivity. These microenvironmental modifications manifest in macroscopic property changes. D_2_O‐containing solutions exhibit lower freezing temperatures than H_2_O‐containing systems due to the more compact HBs network in D_2_O (Figures 3d and S11).^[^ ^30^ ^]^ Remarkably, solutions with a D_2_O: DMF volume ratio exceeding 2:1 remain liquid at ‐75 °C, demonstrating the formation of a robust, reconstructed HB network through enhanced D_2_O‐DMF interactions.

The electrostatic effect between Al^3+^ and SO_4_^2−^ is too strong to break by water, allowing the high lattice energy and a very low solubility of dehydrated aluminum sulfate. Aluminum sulfate hydrate is a better choice to get a solution with a certain Al^3+^ concentration. The addition of incidental H_2_O complicates the solution. And H of H_2_O would be superseded by D to exist in the form of HOD or D_2_O.^[^ ^31^ ^]^ The H/D exchange also occurs between the D_2_O and DMF with a relatively slow rate, Figure 3e. The exchange process will eventually reach a dynamic balance. The substitution of D and the anchoring effect by DMF elevate the energy barrier of D transfer to confine the ZPM and tunneling process (Figure 3f). With these effects, a stronger D‐bond bridged by localized D gets less likely to disintegrate. These synergistic effects stabilize the D‐bond network, reducing its susceptibility to disintegration and significantly enhancing the electrochemical stability of the solution.

### The Solvation Structure of Al^3+^

Vehicular transport mechanisms shed light on another route for HAT. Studies revealed a dynamic octahedral solvation structure of [Al(H_2_O)_5_OH]^2+^ in Al(OTf)_3_ solutions, without Al‐OTf contact ion pairs, even at elevated concentrations.^[^ ^2^ ^]^ Under applied potentials, the directional migration of Al^3+^ facilitates the transfer of solvated H_2_O or D_2_O, forming a “H/D‐down” continuous network on the Al electrode surface that promotes water decomposition.^[^ ^32^ ^]^ By contrast, SO_4_^2−^ participates in the Al^3+^ solvation shell in the sulfate system. Radial distribution function (RDF) analysis predicts the average distribution of adjacent molecules and ions around Al^3+^. All systems exhibit a coordination number of 6 within 0.3 nm of Al^3+^. Statistical analysis reveals that the first solvation shell of Al^3+^ in H_2_O/0DMF consists of 3.312 H_2_O and 2.692 SO_4_^2–^ (Figure S12a), whereas D_2_O /0DMF contains 3.265 D_2_O and 2.737 SO_4_^2–^ (Figure S12b). DMF incorporation contracts the coordination numbers of H_2_O (3.208), D_2_O (3.201), and SO_4_^2−^ (2.568 for H_2_O/0DMF, 2.552 for D_2_O/0DMF), with 0.230 and 0.251 DMF molecules entering the first solvation shell for H_2_O/0DMF and D_2_O/0DMF, respectively (Figures S12c and 4a). Suppression of NQEs modifies the Al^3^⁺ solvation structure by reducing water activity. Traction of DMF on water molecules increases the Al^3+^‐H_2_O/D_2_O distance, as visualized in MD simulation snapshots (Figures S12d–f and 4b). The dynamic behavior of components induces structural variability of Al^3+^, upshifting configurational entropy. This enhanced disorder further confirms the reorganization of HBs networks.

**Figure 4:**
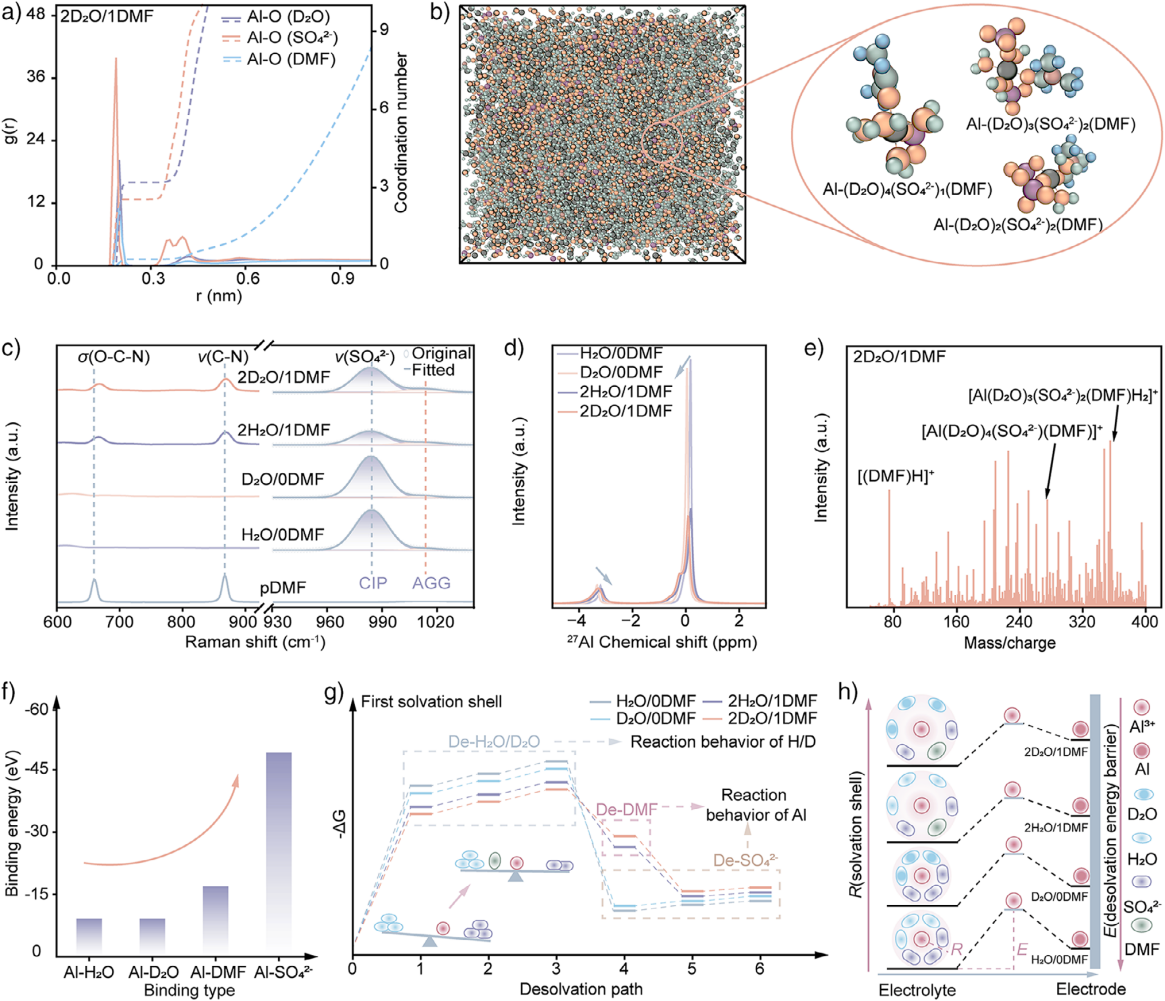
Theoretical and experimental analysis of Al^3+^ solvation structures in different electrolytes. a) RDFs and corresponding coordination numbers for 2D_2_O/1DMF. b) MD simulation snapshots of 2D_2_O/1DMF. c) Raman spectra highlighting DMF and SO_4_^2−^ signatures in different solutions. d) ^[^ ^27^ ^]^Al NMR of different solutions. e) HRMS of ionic species in of 2D_2_O/1DMF. f) Binding energy between Al^3+^ and surrounding species. g) Stepwise desolvation path for different solvated Al^3+^ complexes during the plating process. h) Scheme of solvation shell and their desolvation processes.

Raman spectra were employed to analyze the solvation structure, revealing distinct O─C─N shear [σ(O─C─N), 660 cm^−1^] and C─N stretching [ν(C─N), ∼868 cm^−1^] modes of DMF (Figures 4c and S13).^[^ ^21^ ^]^ The σ(O─C─N) peak exhibits a positive shift due to interactions between the oxygen active site and surrounding components. And clear evidence of contact‐ion pairs (CIPs) and aggregates (AGG) was observed in all solutions.^[^ ^33^ ^]^ This structural configuration is further supported by ^27^Al NMR spectroscopy (Figures 4d and S14). Peaks at 0 ppm are commonly put down to Al(H_2_O)_6_^3+^ with shoulder peaks indicating DMF involvement.^[^ ^34^ ^]^ And shifts to ‐3.3 ppm are attributed to SO_4_^2−^ coordination. The shielding effect of DMF on Al^3+^ leads a negative shift, while it could diminish the shielding effect of SO_4_^2−^ on Al^3+^. It is reflected in the opposite trends for the two peaks of ^27^Al NMR. These observations confirm the existence of Al‐(D_2_O)*_x_* (SO_4_^2−^)*_y_* (DMF)*_z_* complexes. High‐resolution mass spectrometry (HRMS) in positive ion mode provided additional structural insights (Figures 4e and S15). Signals at a mass/charge of 275 and 355 correspond to SO_4_^2−^ and DMF‐containing complexes, respectively. Ion fragments with characteristic isotope patterns were also detected owing to the electrospray ionization, collectively demonstrating significant changes in Al^3+^ coordination environments.^[^ ^35^ ^]^

The binding energies of Al‐H_2_O and Al‐D_2_O are identical (‐9.08 eV) due to the equivalent electronic structures of H and D (Figure 4f). In comparison, the Al‐SO_4_^2−^ interaction exhibits significantly stronger binding energy (‐49.22 eV) due to electrostatic attraction, while Al‐DMF shows intermediate binding strength (‐16.94 eV). These energy differences critically influence Al^3+^ desolvation behavior, subsequently affecting both HER and Al deposition processes. RDF analysis guided the selection of four representative solvation structures, Al‐(H_2_O)_3_(SO_4_^2−^)_3_, Al‐(D_2_O)_3_(SO_4_^2−^)_3_, Al‐(H_2_O)_3_(SO_4_^2−^)_2_(DMF), and Al‐(D_2_O)_3_(SO_4_^2−^)_2_(DMF), for desolvation energy and ESP calculations. The results reveal consistent desolvation energies of ‐14.46 eV for SO_4_^2−^‐rich complexes and ‐2.75 eV for DMF‐containing structures. And DMF also could redistribute the electron density of the complexes to regulate the activity of H_2_O and D_2_O (Figure S16). Besides, the slow and energetically demanding de‐SO_4_^2−^ process emerges as the rate‐limiting step for Al^3+^ electroreduction (Figure 4g). This explains the predominant HER over Al deposition in sulfate solutions during electrodeposition. DMF plays a crucial balancing role by expanding the solvation shell radius (Figure 4h), thereby weakening Al^3+^‐SO_4_^2−^ interactions. Although de‐H_2_O/D_2_O processes preferentially, the restraining effect of DMF and D‐bonds inhibit their decomposition by stronger. The KIE further reduces reaction rates. Collectively, strengthened D‐bonds in the solvation shell, DMF‐mediated structural modifications, and isotope effects create favorable conditions for efficient Al^3+^ electroreduction.

### Investigations of HER and Al Behavior

**Figure 5:**
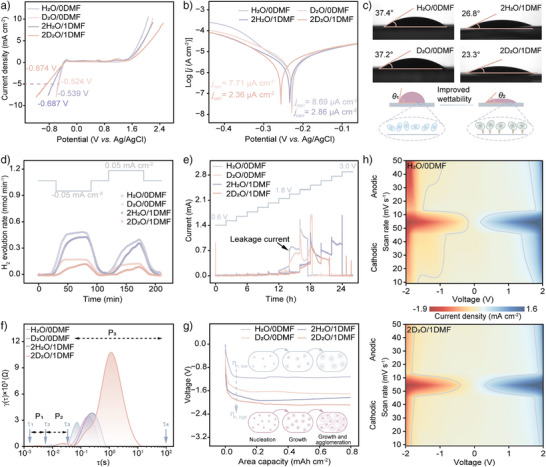
Electrochemical stability of electrolyte and its influence on Al electrode. a) Electrochemical stability windows of various electrolyte. b) Tafel polarization curves for Al electrode. c) Contact angles measurements between Al electrode and electrolyte. d) In situ hydrogen evolution monitoring with corresponding time‐voltage profiles. e) Potentiostatic intermittent test for Al electrode. f) DRT analysis from EIS. g) Electrodeposition overpotential of Al at 0.05 mA cm^−2^. h) Contour plots of CV patterns for Al electrode at different scan rates.

The addition of DMF minimally affected electrolyte pH but significantly altered electrical conductivity (Figure S17), reflecting enhanced intermolecular interactions. Linear sweep voltammetry (LSV) with a platinum working electrode revealed similar HER onset potentials but distinct oxygen evolution reaction (OER) thresholds across electrolyte systems (Figure 5a). Compared to H_2_O/0DMF, the hydrogen evolution potential shifts cathodically from ‐0.524  to ‐0.874 V at 5 mA cm^−2^ for 2D_2_O/1DMF, corresponding to an overpotential increase of 350 mV. A consistent trend was also observed in the LSV profiles of the Al electrode (Figure S18). Both HER and OER overpotentials augmented with current density and DMF content, demonstrating effective HER inhibition through suppressed NQEs. Tafel analysis provided corrosion potentials and current densities (Figure 5b). The corrosion potential shifted negatively to ‐0.26 V, indicating reduced reactivity. And the corrosion current density decreased from 8.69 µA cm^−2^ (H_2_O/0DMF) to 7.71 µA cm^−2^ (D_2_O/0DMF) and further to 2.36 µA cm^−2^ (2D_2_O/1DMF), reflecting slower corrosion rates. The high coincidence of anodic branches contrasted with suppressed cathodic branches, implying selective inhibition of cathodic HER for DMF without significantly affecting anodic Al dissolution. Contact angle measurements embodied an improved wettability, with 2D_2_O/1DMF exhibiting a 23.3° on Al (Figure 5c). This reduced surface tension enhances Al^3^⁺ transport kinetics, potentially lowering the energy barrier for Al nucleation.

Differential electrochemical mass spectrometry (DEMS) was executed to in situ monitor the HER (Figure 5d). The results prove a substantial diminution in HER rate for 2D_2_O/1DMF under both open‐circuit conditions and applied current, clearly specifying that suppressed NQEs effectively inhibit HER in both chemical and electrochemical processes. To evaluate high‐voltage stability, potentiostatic intermittent tests were performed on Al electrodes in different electrolytes at a cut‐off voltage of 3.0 V (Figure 5e). Leakage currents emerged at 2.0 V for H_2_O/0DMF and 2.2 V for D_2_O/0DMF, while 2H_2_O/1DMF and 2D_2_O/1DMF systems delivered leakage currents only above 2.4 V. This suppressed HER not only ameliorates electrode stability but also facilitates efficient Al^3+^ electroreduction.

Electrochemical impedance spectroscopy (EIS) coupled with distribution of relaxation time (DRT) analysis revealed insights into electrode reaction dynamics (Figures S19 and 5f). While suppressed NQEs scarcely alter the fundamental reaction mechanism, as evidenced by consistent three‐peak DRT profiles, they significantly influenced interfacial processes. Peaks P1 and P2 correspond to ohmic impedance and electrical double‐layer relaxation at the electrode‐electrolyte interface, respectively. And P3 represents charge transfer resistance (Rct).^[^ ^36^, ^37^ ^]^ Notably, DMF increased both Rct and relaxation time for P3, suggesting strong DMF‐electrode interactions. This interaction profoundly affects Al nucleation (η_n_) and growth (η_g_) overpotentials (Figure 5g). The continuous processes of nucleation, growth, and agglomeration are governed by sluggish desolvation and DMF‐mediated interactions. And the complicated electrodeposition process and interface characteristics also lead to the non‐uniform nucleation dynamics. In H_2_O/0DMF, a low deposition overpotential of ‐1.1 V allows uneven nucleation and wanton growth, with fresh Al surfaces accelerating HER. Conversely, 2D_2_O/1DMF exhibits higher deposition overpotentials (‐2.1 V), yielding small and dense Al nuclei that form uniform electrodeposited layers. Cyclic voltammetry (CV) profiles (Figure S20) and contour plots (Figures 5h, S21) further demonstrate these effects. The substantial suppression of water splitting peaks in 2D_2_O/1DMF indicates effective HER inhibition. Improved peak symmetry confirms enhanced Al plating/stripping reversibility, demonstrating that strengthened D‐bonds and optimized solvation structures effectively reduce water activity while ameliorating Al deposition behavior.

### Reversible Al Plating/Stripping Behavior

**Figure 6:**
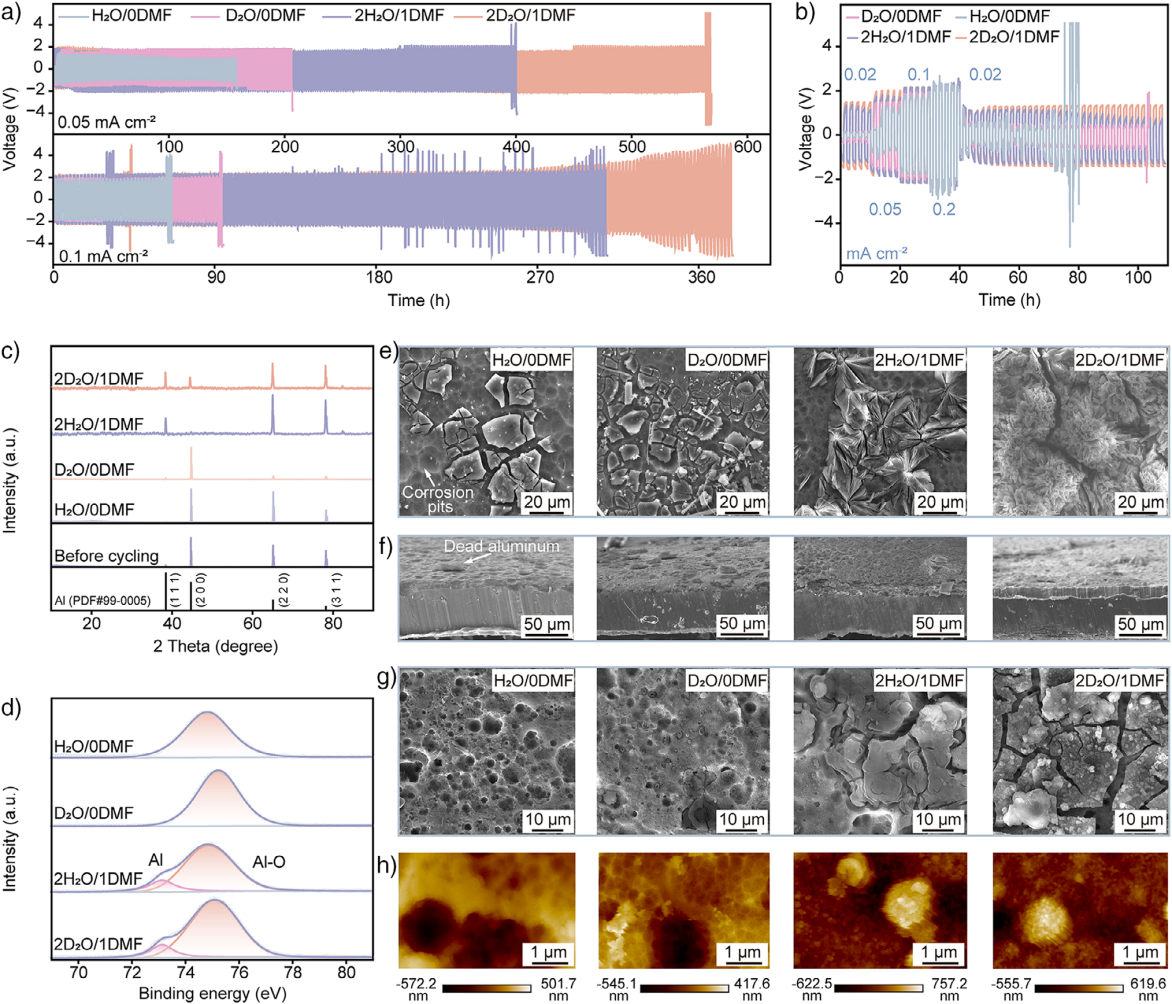
Electrochemical performance of Al anodes and their composition and morphological evolutions. a) Long‐term cycling stability of Al plating/stripping at 0.05 and 0.1 mA cm^−2^. b) Voltage profiles of symmetric cells under progressively upshifting current densities. c–f) XRD patterns (c), Al 2p XPS spectra (d), SEM images (e), SEM images of the cross‐sections (f) for anode after cycling at 0.05 mA cm^−2^. g,h) SEM (g) and (h) AFM images of electrode after electrodeposition for 20 h at 0.05 mA cm^−2^.

To validate the efficacy of the electrolyte in enabling reversible Al behavior, we conducted plating/stripping tests in both symmetric and asymmetric cell configurations. The 2D_2_O/1DMF electrolyte demonstrated exceptional stability, supporting prolonged Al plating/stripping cycles (Figure 6a). Al||Al symmetric cells achieved remarkable lifespans of 569 h at 0.05 mA cm^−2^ and 379 h at 0.1 mA cm^−2^ in 2D_2_O/1DMF, albeit with higher voltage hysteresis (1.6  and 2.3 V, respectively) due to suppressed NQEs (Figure S22). In contrast, H_2_O/0DMF cells exhibited unstable cycling after 158 h (0.05 mA cm^−2^) and 62 h (0.1 mA cm^−2^), despite lower hysteresis (1.1  and 1.8 V, separately). Systematic evaluation of D_2_O:DMF ratios revealed optimal performance at 2:1 (Figure S23). Excessive DMF compromised cell lifetime through increased H/D exchange and reduced conductivity, generating detrimental H_2_O. Battery thickness measurements confirmed these observations (Figure S24). The cell with H_2_O/0DMF experienced severe gas evolution and 24.9% thickness expansion, while 2D_2_O/1DMF maintained structural integrity through effective HER suppression. Al||Ti asymmetric cells further demonstrated enhanced Al behavior in 2D_2_O/1DMF, achieving coulombic efficiencies (CE) of 87.24% (30 cycles) and 95.56% (100 cycles) at 0.05 mA cm^−2^ (Figures S25, S26). This improvement reflects stable aluminum nucleation and growth kinetics. In contrast, H_2_O/0DMF cells exhibited rapid failure. Rate performance tests (Figure 6b) confirmed superior performance in 2D_2_O/1DMF, with stable voltage hysteresis maintained even at 0.2 mA cm^−2^, while H_2_O/0DMF cells failed within 80 h. These electrochemical improvements stem from HER suppression and optimized Al^3+^ solvation structures achieved through controlled NQEs, demonstrating the critical role of D effects and molecular interactions in enabling reversible Al electrochemistry.

X‐ray diffraction (XRD) analysis of Al electrodes before and after cycling suggests preferential induction of DMF for (111) crystal plane growth (Figures 6c and S27). It could be attributed that the DMF preferentially adsorbs onto (200) facets via its carbonyl group (C = O), passivating the Al (200) growth. And there is absent for other impurity phases in all samples. X‐ray photoelectron spectroscopy (XPS) confirmed the presence of Al‐O bonds (Figure 6d), consistent with spontaneous aluminum oxidation.^[^ ^38^, ^39^ ^]^ Obviously, shoulder peaks related to metallic Al appeared in electrodes after cycling in 2H_2_O/1DMF and 2D_2_O/1DMF systems. Scanning electron microscopy (SEM) provided insights into morphological evolution (Figures S28 and 6e). H_2_O/0DMF electrodes developed irregular lumpy deposits with underlying honeycomb corrosion pits after 159 h at 0.05 mA cm^−2^. D_2_O/0DMF systems showed smaller, denser deposits after 207 h. Corrosion‐induced stress concentration initiates crack propagation that critically compromises the structural continuity of electrode surface deposition layers, accelerating interfacial deterioration through localized current focusing. Noteworthily, some of the deposited Al becomes curly and disengages from electrode surface to be “dead aluminum”, which could speed up the corrosion and worsen electrode stability (Figure 6f). In contrast, the electrode exhibited tightly adhered flower‐like deposits with suppressed corrosion after 401 h in 2H_2_O/1DMF. The 2D_2_O/1DMF system demonstrated optimal performance, with dense, smooth deposits and minimal corrosion after 569 h. This vertical growth morphology, characterized by gap‐filling Al nuclei, explains the observed XRD and XPS differences, highlighting the critical role of controlled nucleation in electrode stability.

The constant‐current plating was performed at 0.05 mA cm^−2^ to elucidate electrolyte effects on Al electroreduction, with the initial hour monitored via in situ optical microscopy (Figure S29). Copious bubble generation accompanied the formation of highly irregular and porous deposits in H_2_O/0DMF. Progressive plating led to deposit detachment due to bubble‐induced disturbances, exposing fresh surfaces susceptible to corrosion and HER. Driven by higher activation energies, the localized Al deposition occurred at depression edges after 20 h (Figure 6g). Dendritic growth was eliminated in D_2_O/0DMF, promoting more uniform deposit attachment and corrosion pit filling, as also confirmed by atomic force microscopy (AFM) (Figures 6h and S30). Introducing DMF further improved morphology, replacing loose “dead aluminum” and deep pits with fine and uniformly distributed particles. The 2D_2_O/1DMF (Rq = 137 nm) system exhibited superior deposit uniformity compared to 2H₂O/1DMF (Rq = 189 nm), with controlled crack propagation and consistent layer thickness. These morphological differences directly correlate with the cycling stability of the electrode. The suppressed NQEs in 2D_2_O/1DMF effectively inhibit HER, enabling stable Al plating/stripping and significantly extending cell lifetime through maintained interface stability.

## Conclusion

In summary, we have developed a HB reinforcement strategy through modulation of NQEs for hydrogen, enabling precise control of HER inhibition and Al deposition behavior. Multiscale assessments reveal that suppressed NQEs in mixed electrolyte systems strengthen intra‐ and intermolecular D‐bonds while optimizing Al^3^⁺ solvation structures. This approach simultaneously enhances electrolyte stability by reducing water activity via both thermodynamic and kinetic mechanisms, effectively suppressing competitive HER while promoting efficient Al plating. Comprehensive morphological analysis demonstrates stable and efficient Al reduction. The well‐defined solvation structures customize the Al nucleation and growth behavior, boosting the spatially uniform distribution of Al nuclei and a dense and smooth plating layer. The 2D_2_O/1DMF electrolyte exhibits a 350 mV increase in HER overpotential at 5 mA cm^−2^ compared to H_2_O/0DMF. Remarkably, symmetric cells achieve extended cycling lifetimes of 569 h (0.05 mA cm^−2^) and 379 h (0.1 mA cm^−2^) in 2D_2_O/1DMF, representing 3.6‐ and 6.1‐fold improvements over H_2_O/0DMF, respectively. These findings provide fundamental insights into HBs behavior from a quantum behavior regulation and establish a new exemplification for achieving highly reversible Al redox in aqueous sulfate electrolytes.

## Conflict of Interests

The authors declare no conflict of interest.

## Supporting information



Supporting Information

## Data Availability

The data that support the findings of this study are available from the corresponding author upon reasonable request.
