# Supplementary material for: Nuclear Quantum Confinement Enables Robust Deuterium Bonds for Highly Reversible Aluminum Anodes
Source: Angew Chem Int Ed Engl. 2025 Jul 9;64(35):e202508422. doi: 10.1002/anie.202508422 (PMC12377455; doi:10.1002/anie.202508422)
Supplement: Supplementary file 1 — Supporting Information [file ANIE-64-e202508422-s001.docx]

**Supporting Information**

**Nuclear Quantum Confinement Enables Robust Deuterium Bonds for Highly Reversible Aluminum Anodes**

Hao Cheng^1, 2^, Yao Lu^1, 3^, Zheng Li ^4^, Zibo Chen^1, 2^, Chao Chen^1, 3^, Xinyi Li^1, 2^, Hailin Yu^1, 2^, Adham Hashibon^5^, Zhongliang Tian^1, 2, 3*^, Guanjie He^6*^

^1^ School of Metallurgy and Environment, Central South University, Changsha 410083, China. ^2^ National Engineering Research Centre of Low-carbon Nonferrous Metallurgy, Changsha 410083, China. ^3^National Energy Metal Resources and New Materials Key Laboratory, Changsha 410083, China. ^4^School of Materials Science and Engineering, Hunan University of Science and Technology, Xiangtan 411201, China. ^5^Institute for Materials Discovery, University College London, 107 Roberts Building, London, WC1E 7JE, UK. ^6^Department of Chemistry, University College London, 20 Gordon Street, London, WC1H 0AJ, UK.

**Methods**

**The preparation of the electrolyte**

1.37 g of aluminum sulfate hydrate (Al_2_(SO_4_)_3_·xH_2_O, 98%, Aladdin) was dissolved into 2 mL of purified water or heavy water (D_2_O, 99%, Aladdin) to prepare DMF-free electrolytes designated as H_2_O/0DMF and D_2_O/0DMF, respectively. For DMF-containing electrolytes, the same amount of Al_2_(SO_4_)_3_·xH_2_O was dissolved in mixtures of H_2_O (or D_2_O) and N, N-dimethylformamide (DMF, 99.5%, Sinopharm) at volume ratios of 4:1, 2:1, and 1:1, yielding electrolytes labeled as 4D_2_O/1DMF, 2D_2_O/1DMF (or 2H_2_O/1DMF), and 1D_2_O/1DMF, respectively. All mixtures were homogenized at 60 °C using a magnetic stirrer until fully dissolved.

**Characterizations**

FTIR spectroscopy with attenuated total reflectance (ATR) attachment (Thermo Fisher Scientific Nicolet iN10), Raman spectroscopy with 532 nm excitation (HORIBA LabRAM HR Evolution), and NMR spectroscopy (Bruker 600MHz) were used to characterize and analyze hybrid electrolytes. DSC (Netzsch DSC 200 F3) was adopted to estimate the freezing point. HRMS was performed in positive ion mode (Thermo Fisher Scientific Q Exactive). The Contact angle tests were acquired on the Kruss DSA100 optical contact angle system. Electrode morphology was characterized by SEM (TESCAN) and AFM (Bruker Dimension Icon). Surface chemical composition was analyzed by XRD (SmartLab, Cu Kα radiation) and XPS (Thermo Scientific K-Alpha). In situ optical microscopy of Al plating was conducted using a customized cell with an optical microscope (AOSVI NX30T-HK830).

**Electrochemical measurements**

LSV with a scan rate of 0.01 V s^-1^ was performed using a three-electrode configuration on an electrochemical workstation (CHI660) with a platinum working electrode, platinum counter electrode, and Ag/AgCl reference electrode. The dosage of electrolyte is 10 mL for three-electrode system test. For Al anode, the oxide film on the aluminum surface has been removed by the soaking in the 4M NaOH solution for 2 min. And then the electrode was soaked in 0.02M ZnSO_4_ solution for 6 hours to prevent further oxidation. Tafel analysis with a scan rate of 0.01 V s^-1^ and CV were conducted using the same system, substituting the working electrode with an Al sheet. DEMS measurements were acquired using a QAS100 Li system with argon carrier gas at a flow rate of 1 mL min^-1^. Symmetric Al||Al cells (CR2032 coin cells) were assembled using Al foils (13 mm diameter, 100 μm thickness) as both working and counter electrodes, separated by glass microfiber filters (Whatman, GF/D). Asymmetric Al||Ti cells employed titanium foils of identical dimensions as the working electrode. And the dosage of electrolyte is 100 μL for asymmetric and asymmetric cells. Al||Al cells were conducted using the CHI660 workstation. Potentiostatic intermittent tests, electrodeposition overpotential measurements, and galvanostatic cycling of Al||Al cells, along with coulombic efficiency (CE) tests for Al||Ti cells, were performed using a battery testing system (LAND CT2001A) at 25 °C. Additionally, the range of EIS was set as 10^5^ Hz - 0.01 Hz with an amplitude of 5mV.

**Theoretical calculations**

All DFT calculations were performed using the Gaussian 09 software suite^1^. Initial conformational searches were conducted using the Molclus program integrated with MOPAC v22.0.4, employing the PM6-DH+ method for preliminary structure optimization^2^. Subsequent calculations utilized the B3LYP functional with D3BJ dispersion correction for structural optimization, vibrational frequency analysis, and energy calculations, using the 6-31G* basis set^3^. Binding energies were computed with the M06-2X functional and def2-TZVP basis sets^4^. Electrostatic potential (ESP) analysis was performed using Multiwfn 3.8, with visualization conducted in VMD^5,6^.

**MD simulation**

Classical MD simulations were carried out using the GROMACS 2021.5 package to investigate the mixed solution at the atomic level^7^. Atomic motion was described by Newton’s equations, integrated using the velocity-Verlet algorithm. Initial system configurations were generated using PACKMOL software^8^. The OPLSAA force field was employed for DMF and SO_4_^2-^, while the tip4p model was used for H_2_O and D_2_O^9,10^. The potential parameter of Al was obtained from the reference ^11^. Van der Waals and electrostatic interactions were truncated at 1.2 nm. Systems were first energy-minimized to relax the simulation box, followed by equilibration in the isothermal-isobaric (NPT) ensemble with a 1.0 fs time step at 298.15 K and 1.0 atm, maintained using the Nose-Hoover thermostat and Parrinello-Rahman barostat, respectively. A 10.0 ns NPT equilibration ensured stable box dimensions. RDFs were calculated with a bin size of 0.1 Å. Hydrogen bonds were identified using geometric criteria: O-O distances < 3.5 Å and O-H-O angles < 30°. Hydrogen bond lifetimes were determined from time autocorrelation functions of hydrogen bond persistence.


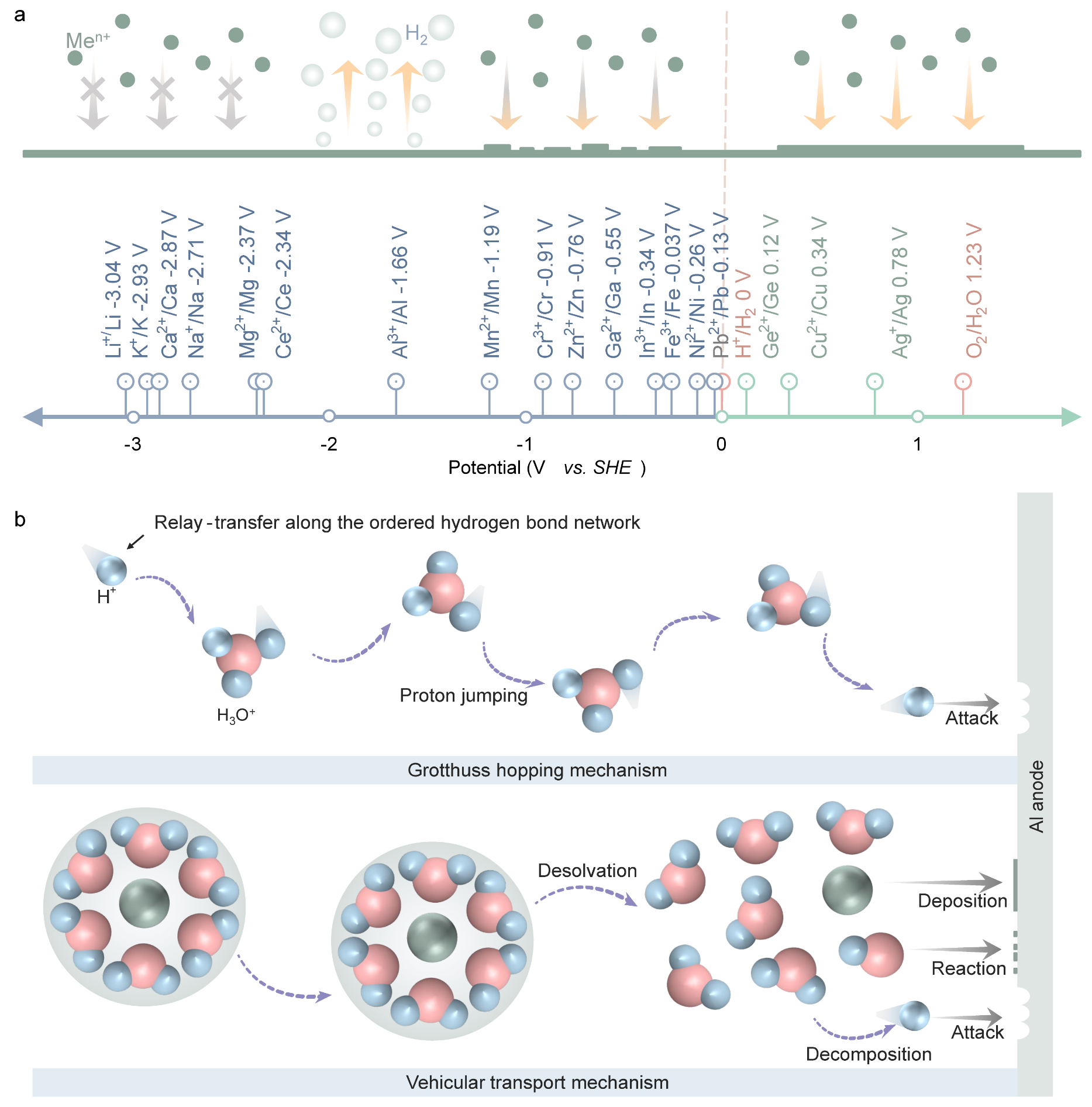


***Figure S1.*** (a) Comparison of redox potentials of different ions. (b) Schematics of the Grotthuss hopping mechanism of proton and vehicular transport mechanism. In aqueous electrolytes, the hydrogen evolution reaction (HER) dominates under potentials below the H^+^/H_2_ redox potential. While overpotentials from mass transport limitations or interfacial effects can enable electroreduction of select metal ions (e.g., Zn²⁺, In³⁺, and Fe³⁺), ions with lower reduction potentials (such as Al³⁺, Mg²⁺, Li⁺, and Na⁺) resist reduction due to their strong metallic character and competing water decomposition. Consequently, molten salt electrolysis has emerged as the primary route for extracting these metals. Thus, controlling HER kinetics is pivotal to enabling efficient electroreduction processes for active metal ions in aqueous systems.


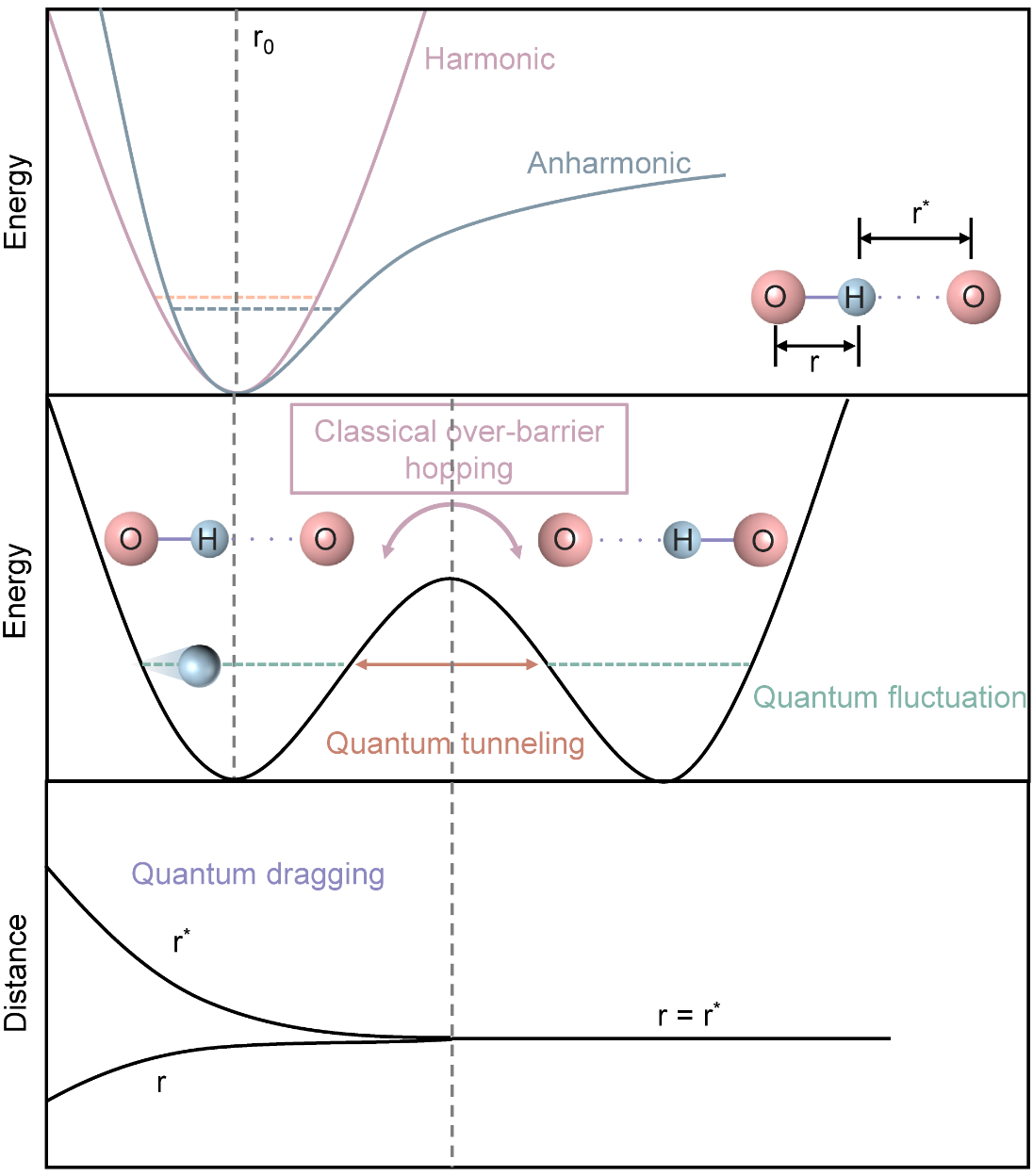


***Figure S2.*** Nuclear quantum effects (NQEs) of hydrogen atom. The quantum nature of hydrogen bonds originates from the quantum dynamics of light hydrogen nuclei, fundamentally governing the structural, dynamic, and macroscopic properties of water. Precise manipulation and quantification of nuclear quantum effects (NQEs) enable effective HER suppression through hydrogen behavior modulation. While classical particles occupy the minimum potential energy state, the Heisenberg uncertainty principle dictates persistent zero-point motion (ZPM) of hydrogen nuclei. In real systems, the anharmonic potential field further displaces hydrogen from its classical equilibrium position, inducing variations in hydrogen bond strength. Classical models describe hydrogen bond-covalent bond transitions as over-barrier hopping processes, whereas quantum tunneling facilitates bond conversion at reduced energy barriers. This quantum behavior, coupled with the dynamic equilibrium maintained by surrounding water molecules, enhances water activity through quantum mechanical properties of hydrogen^12-16^.


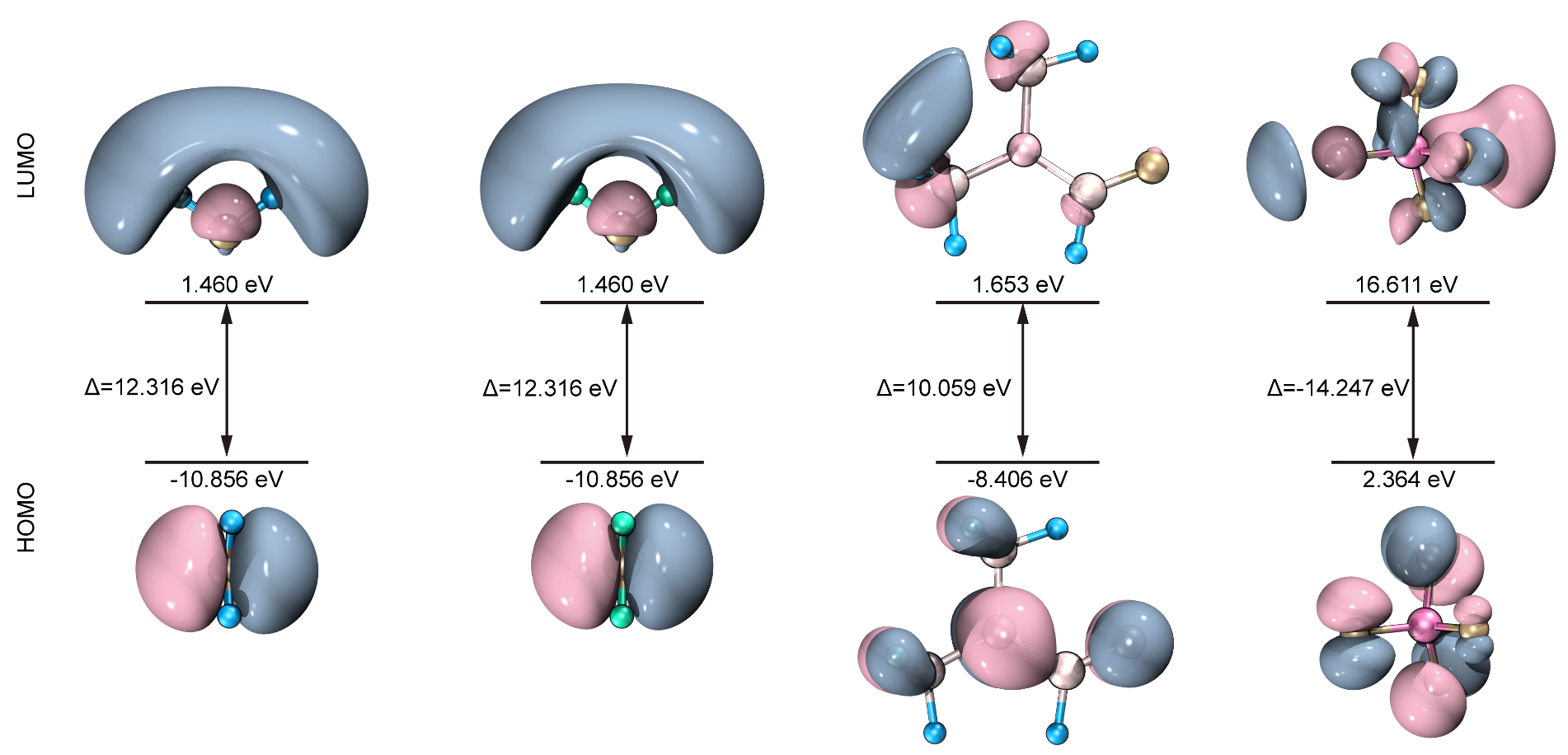


***Figure* *S3.*** Comparison of front orbitals of different molecules.


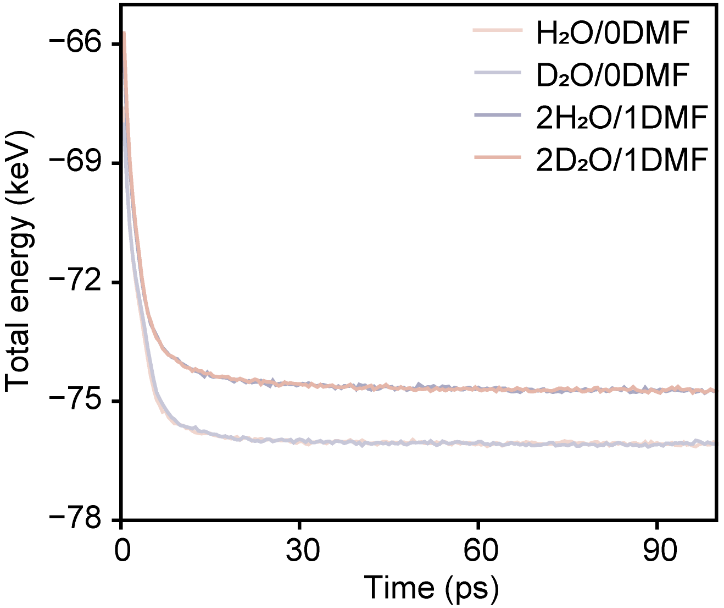


***Figure S4.*** The total energy change of different sysytems during the molecular dynamics (MD) simulation.


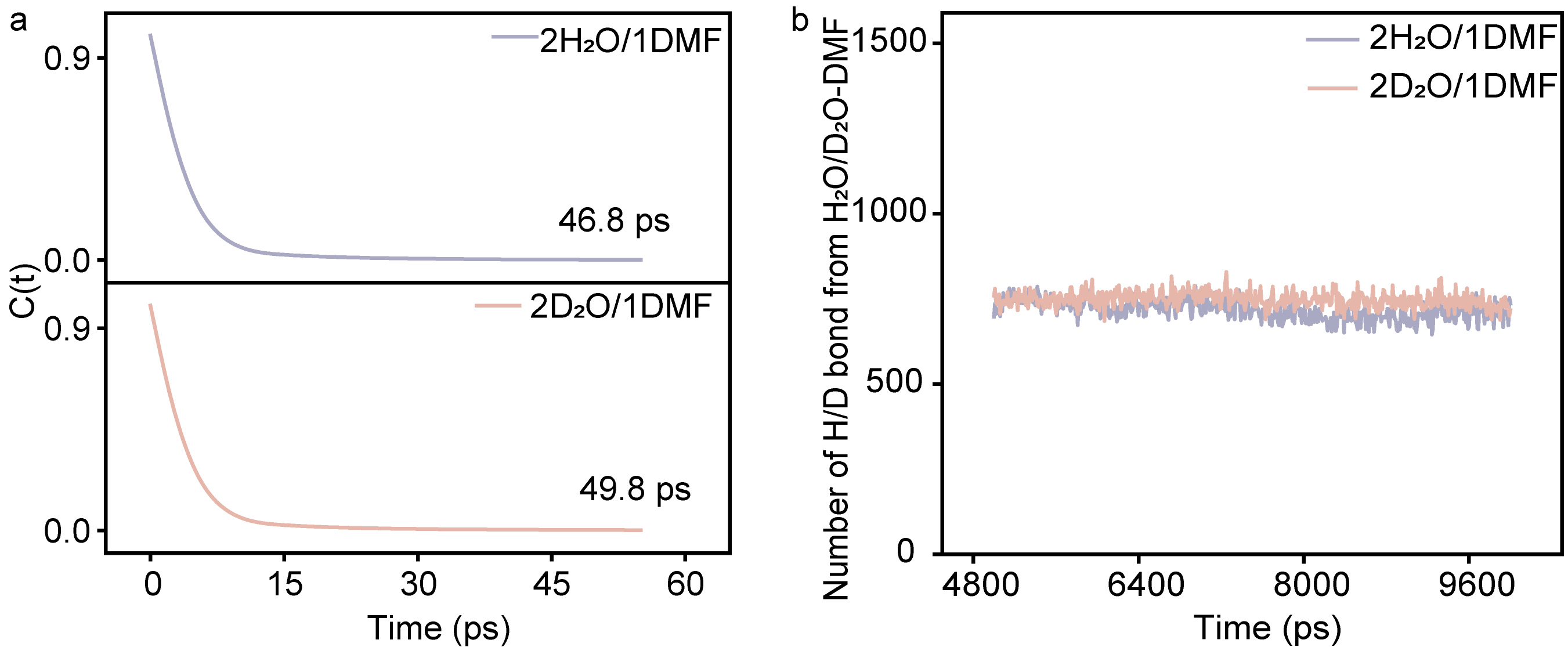


***Figure S5.*** The H/D-bonds lifespan (a) and number (b) between the H_2_O/D_2_O and DMF.


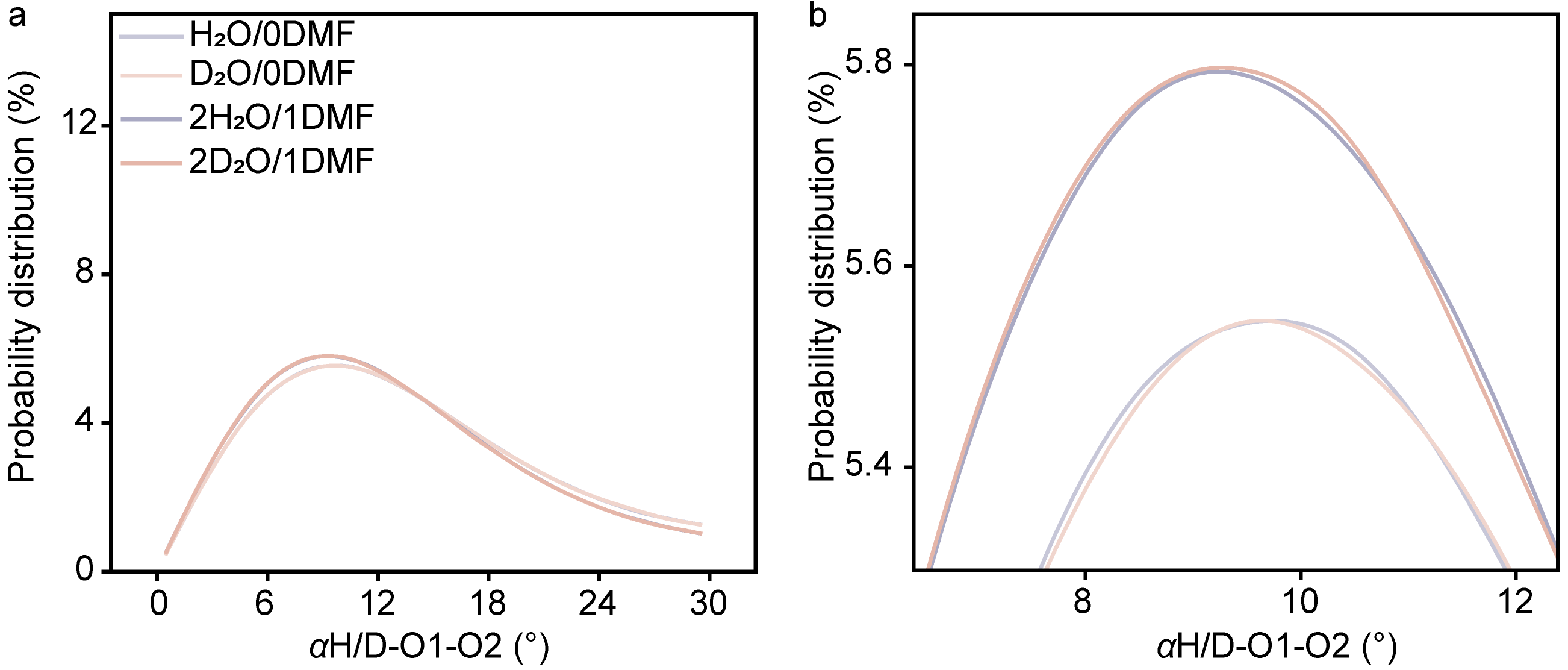


***Figure* *S6.*** Probability distributions of α_H/D-O1-O2_.


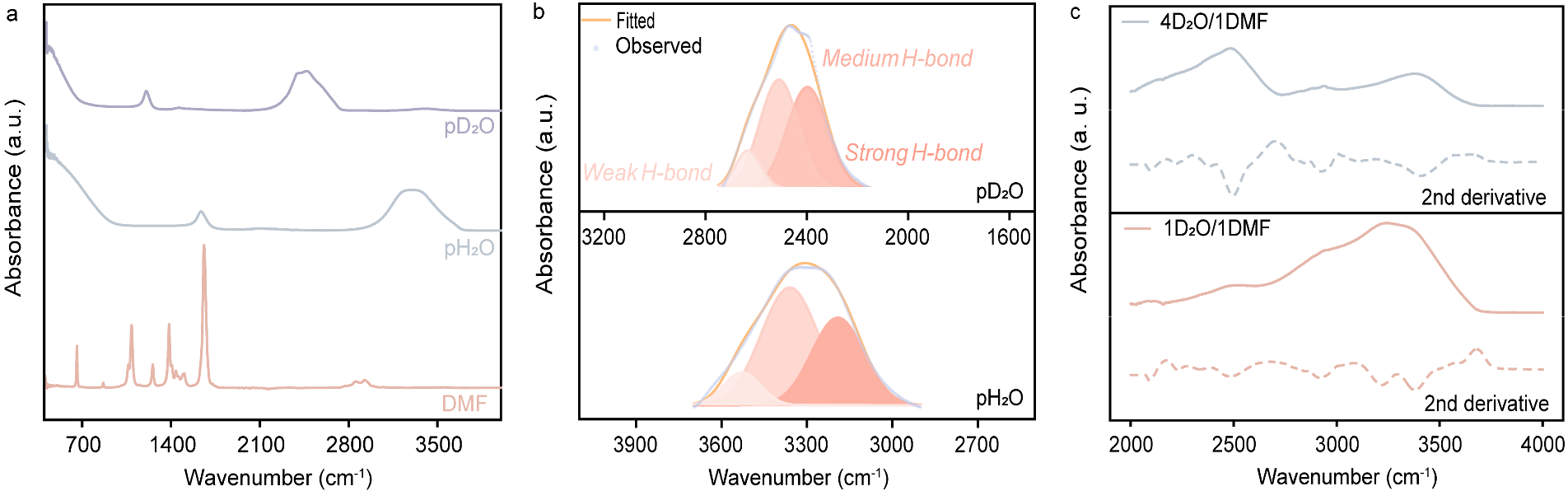


***Figure* *S7.*** (a) Fourier transform infrared (FTIR) spectroscopy for pure substance. (b) Fractional fitting of O-H/O-D stretching vibration mode. (c) FT-IR absorbance and second derivative spectra for 4D_2_O/1DMF and 1D_2_O/1DMF.


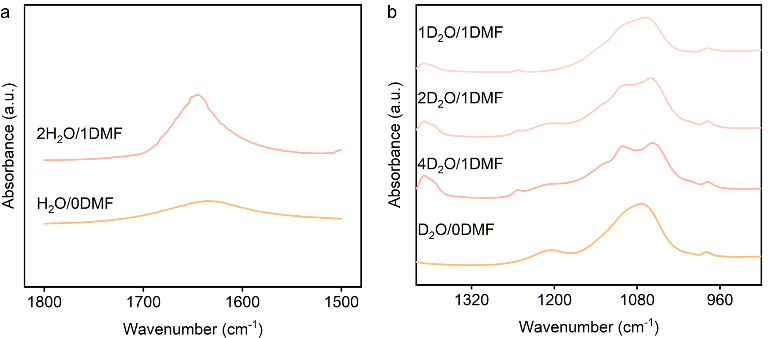


***Figure* *S8.*** FT-IR absorbance for OH (a) and OD(b) bending vibration


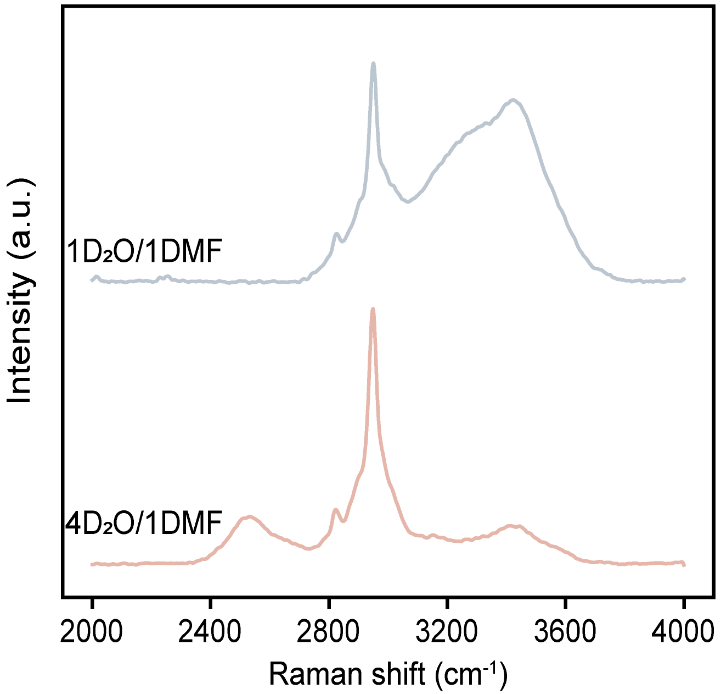


***Figure* *S9.*** Raman spectra for 4D_2_O/1DMF and 1D_2_O/1DMF.


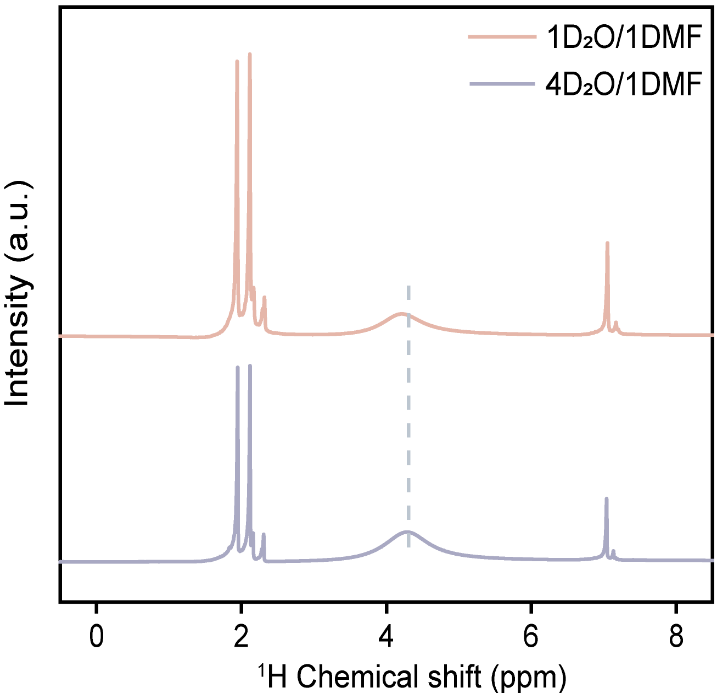


***Figure S10.*** ^1^H NMR spectra for 4D_2_O/1DMF and 1D_2_O/1DMF.


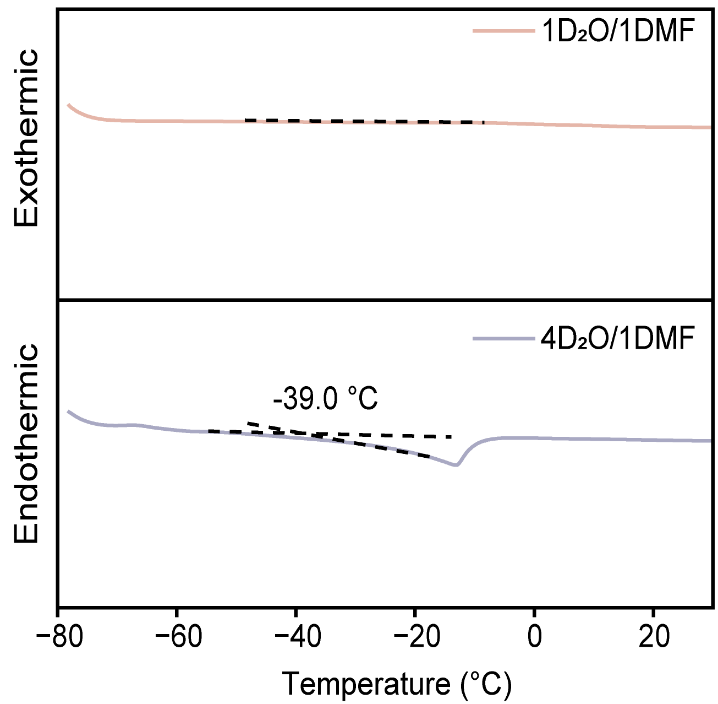


***Figure* *S11.*** DSC data for 4D_2_O/1DMF and 1D_2_O/1DMF.


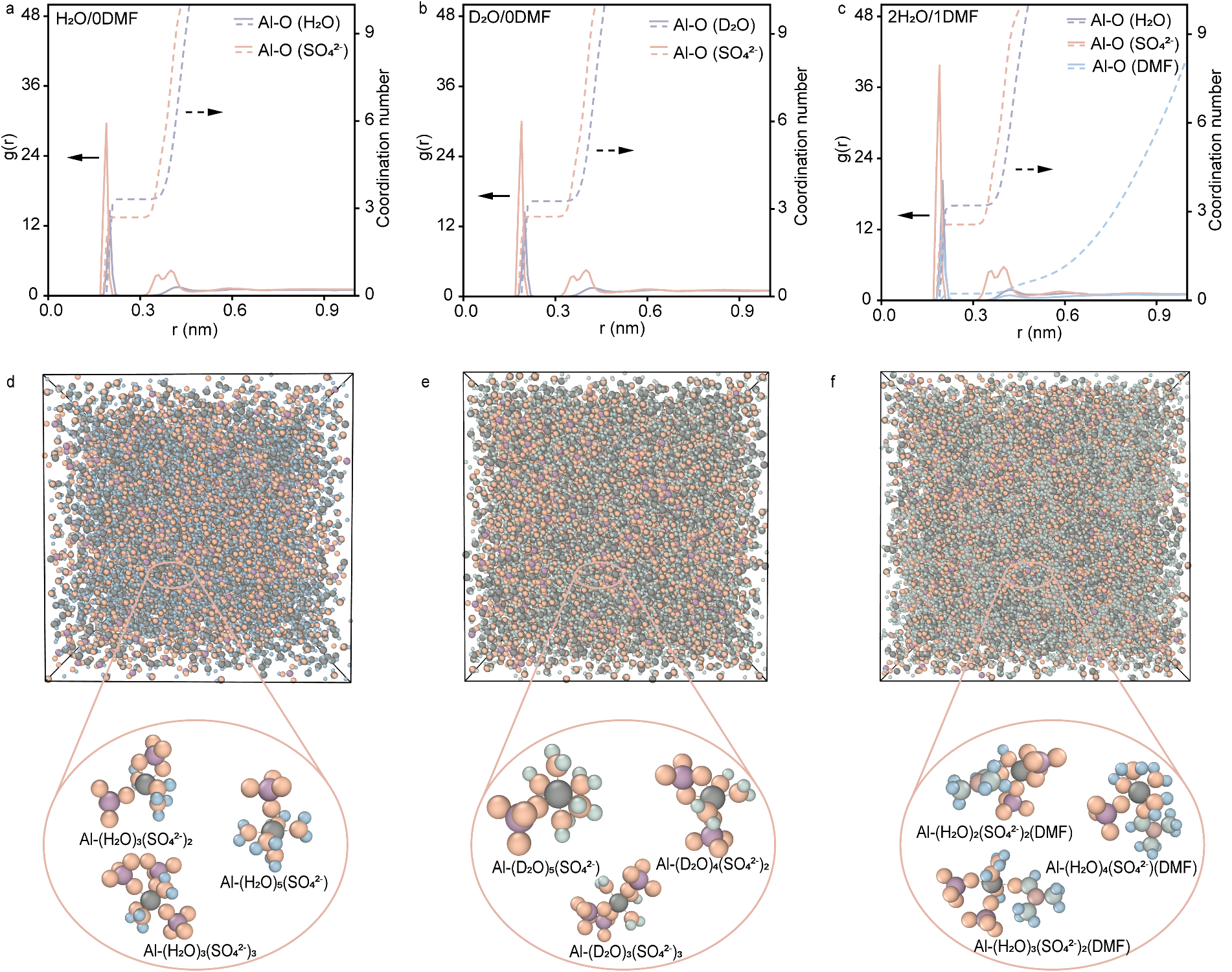


***Figure* *S12.*** Analysis of the solvation structure of the electrolytes. RDFs and corresponding coordination numbers for H_2_O/0DMF (a), D_2_O/0DMF (b) and 2H_2_O/1DMF (c). Snapshots of the MD simulation of H_2_O/0DMF (d), D_2_O/0DMF (e) and 2H_2_O/1DMF (f).


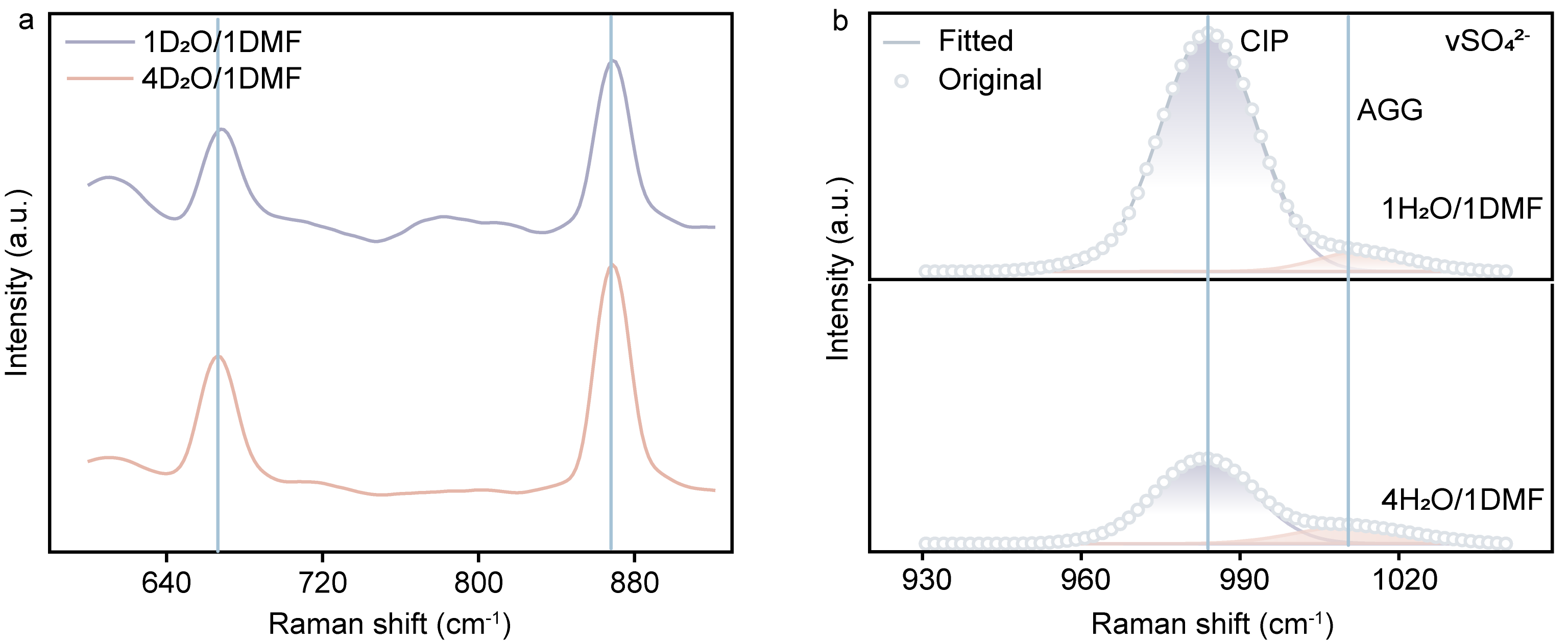


***Figure S13.*** Raman spectra of 4D_2_O/1DMF and 1D_2_O/1DMF for DMF (a) and SO_4_^2-^ (b).


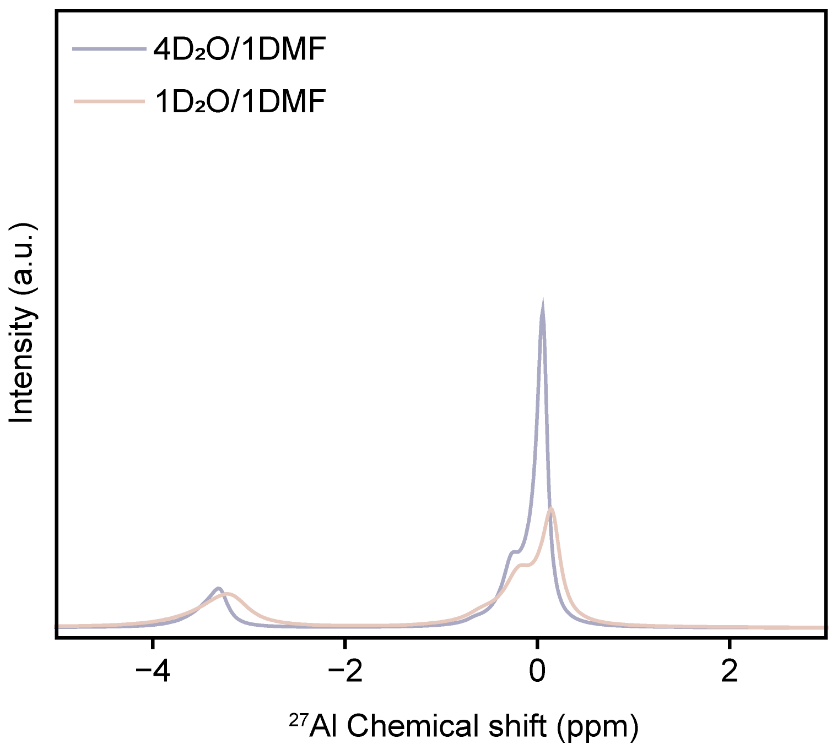


***Figure S14.*** ^27^Al NMR for 4D_2_O/1DMF and 1D_2_O/1DMF.


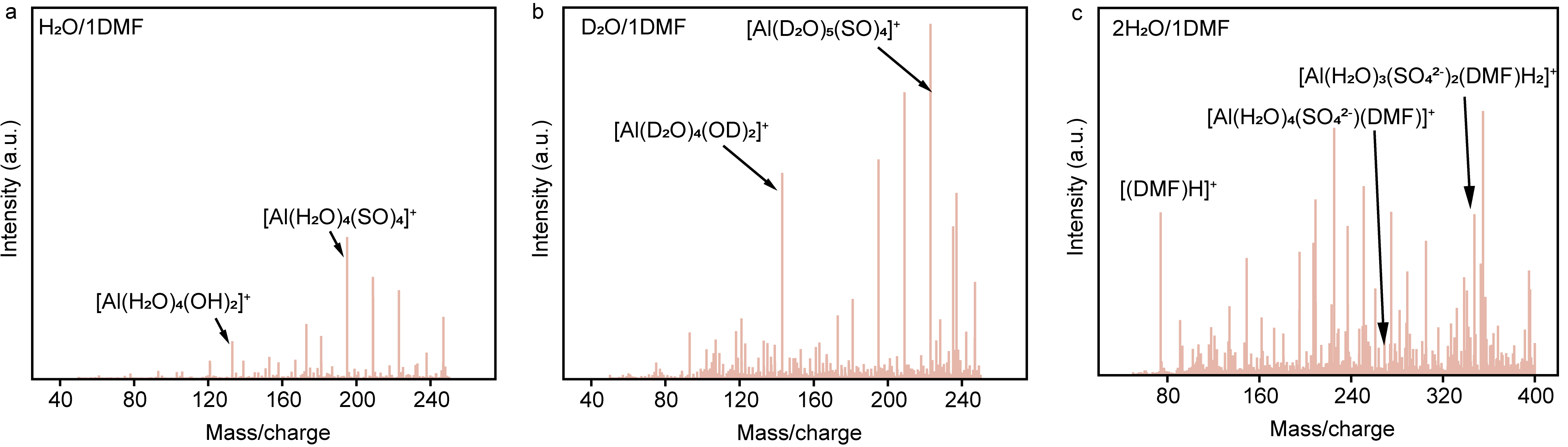


***Figure S15.*** HRMS for 4D_2_O/1DMF and 1D_2_O/1DMF.


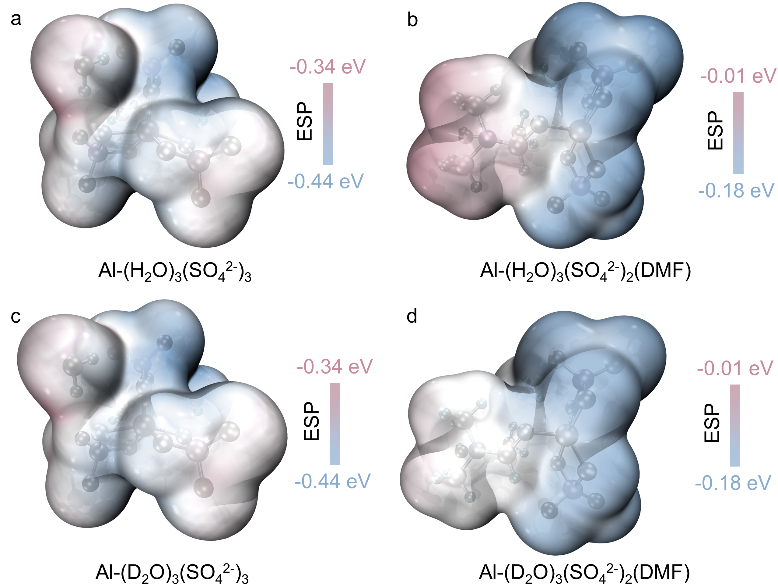


***Figure S16.*** The ESP of different solvation structures.


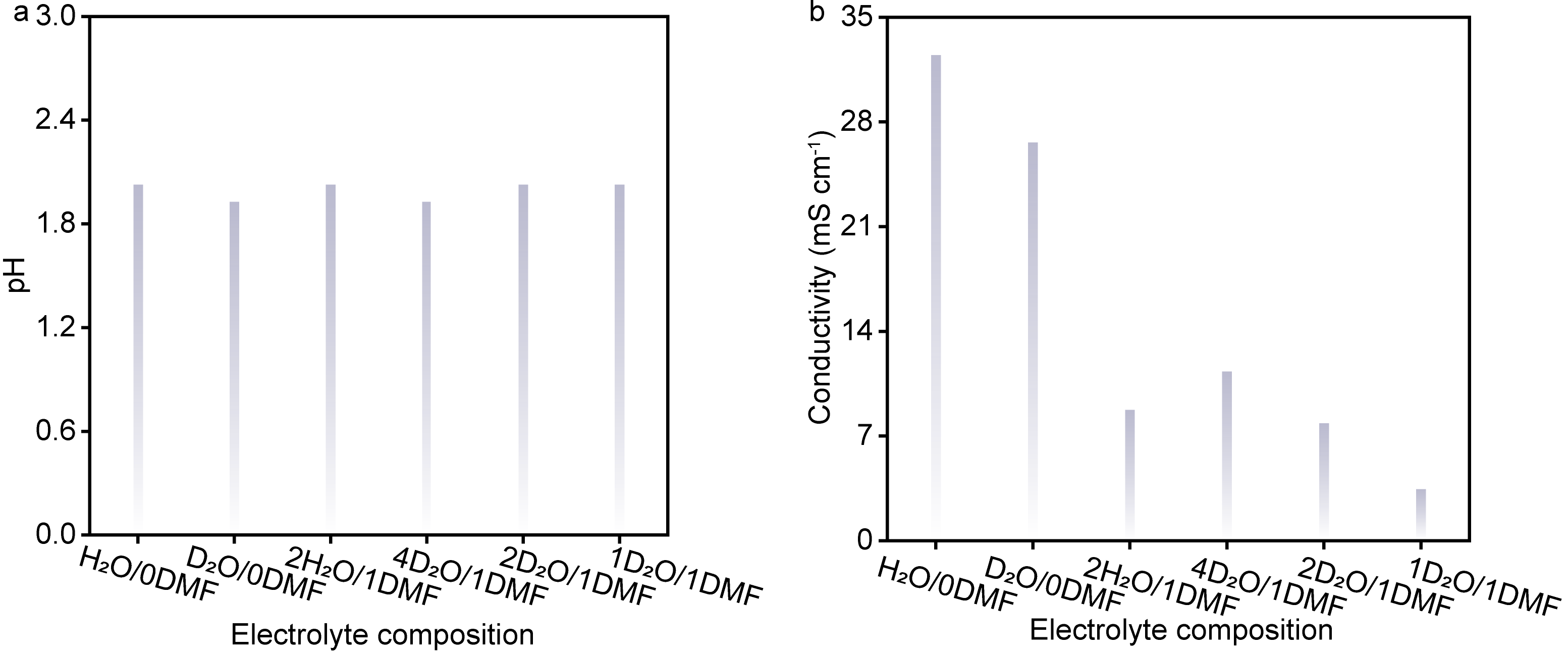


***Figure* *S17.*** Physicochemical properties of the electrolytes. (a) pH. (b) Conductivity.


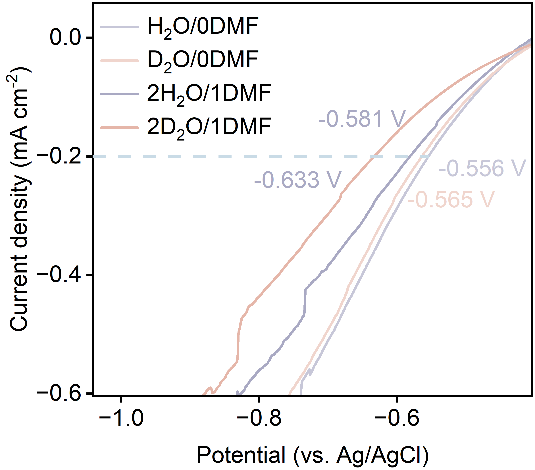


***Figure S18.*** The LSV curves with an aluminum working electrode.


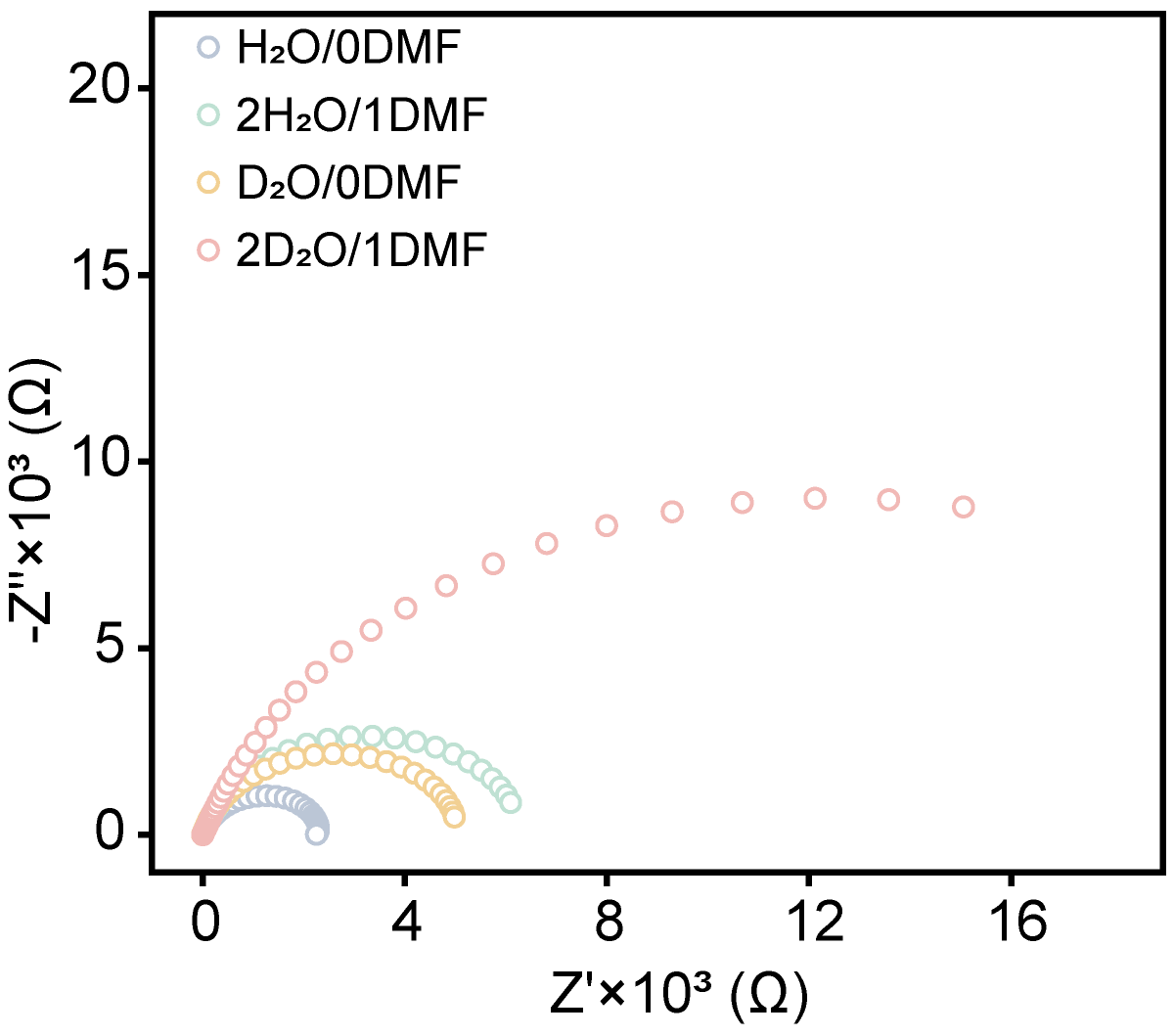


***Figure* *S19.*** Nyquist plots of the Al electrode in various electrolytes.


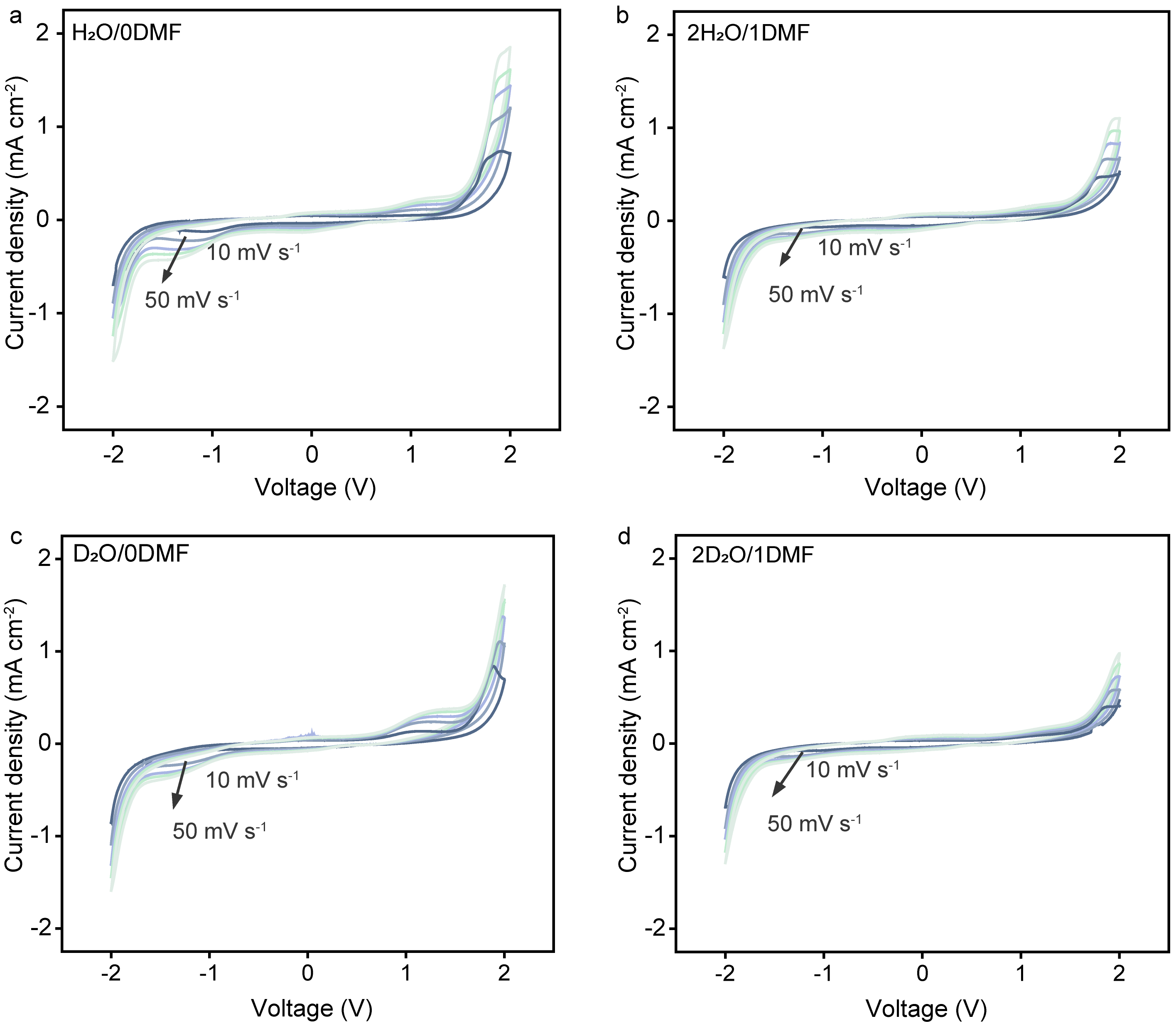


***Figure* *S20.*** CV profiles with different scan rates. (a) H_2_O/0DMF. (b) 2H_2_O/1DMF. (c) D_2_O/0DMF. (d) 2D_2_O/1DMF.


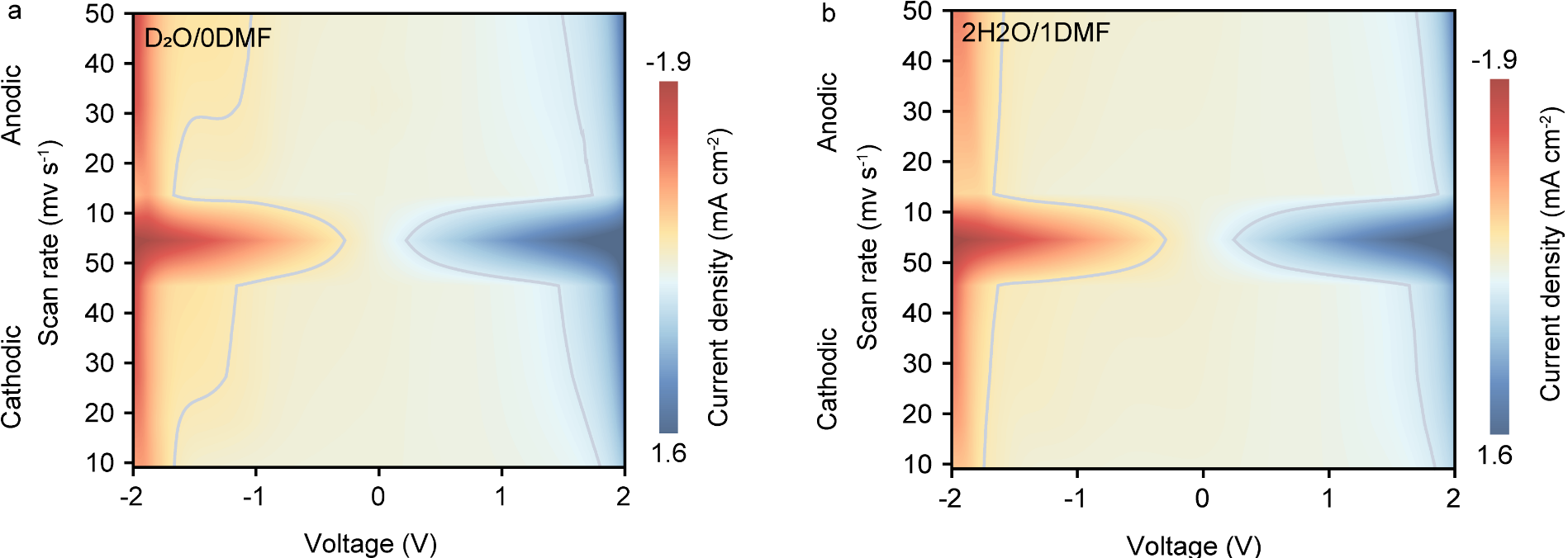


***Figure* *S21.*** Contour plots of CV patterns for Al electrode with different scan rates. (a) D_2_O/0DMF. (b) 2D_2_O/1DMF.


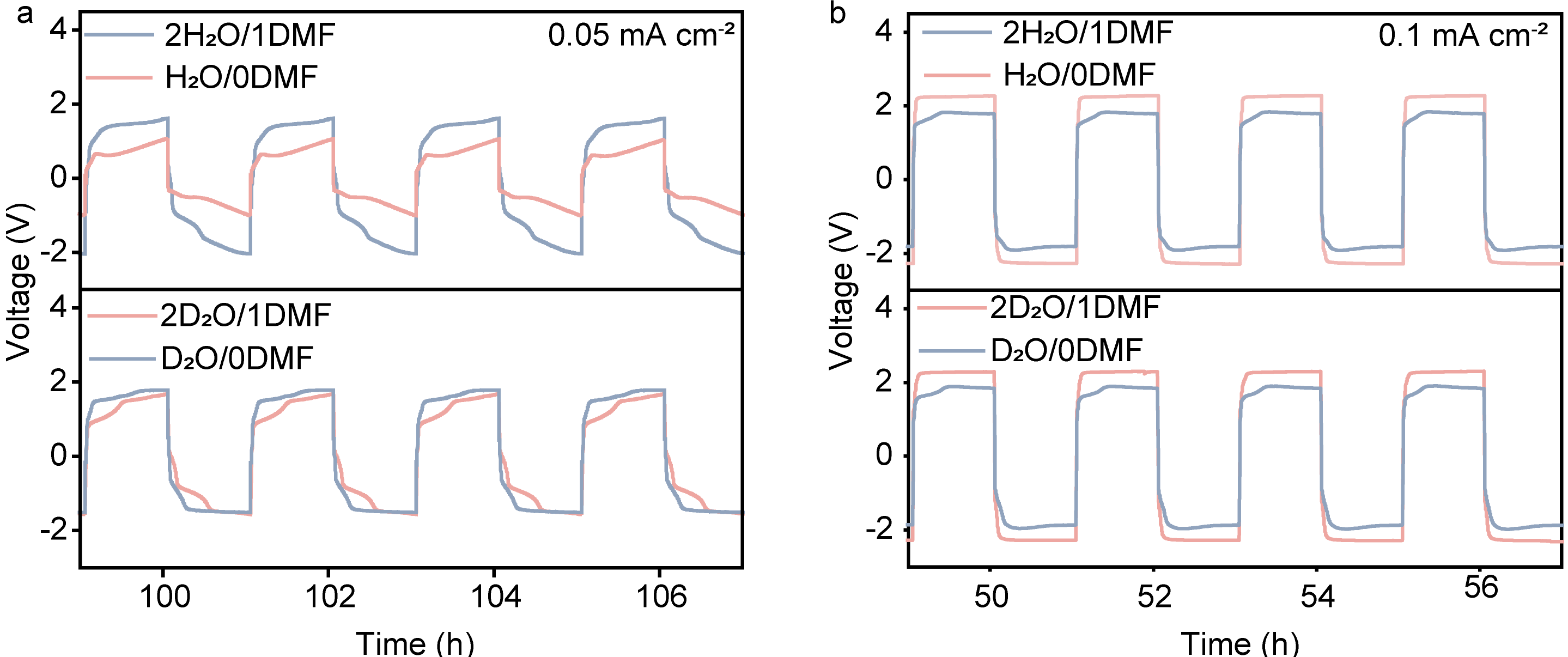


***Figure S22.*** Local enlarged diagram of charge-discharge curves at 0.05 (a) and 0.1 (b) mA cm^-2^.


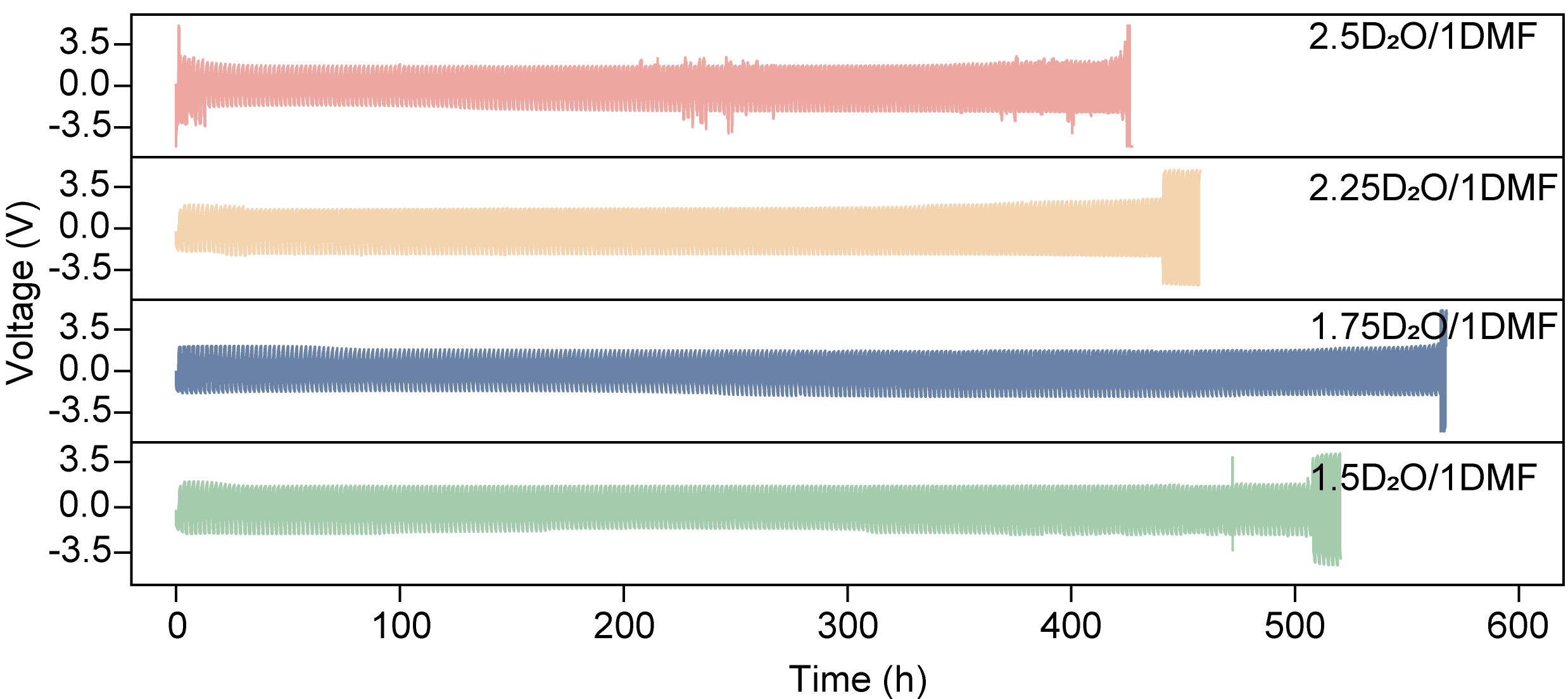


***Figure* *S23.*** Deposition and stripping cyclic stability of the anode at 0.05 and 0.1 mA cm^-2^ in the electrolytes with various volume ratio of D_2_O and DMF.


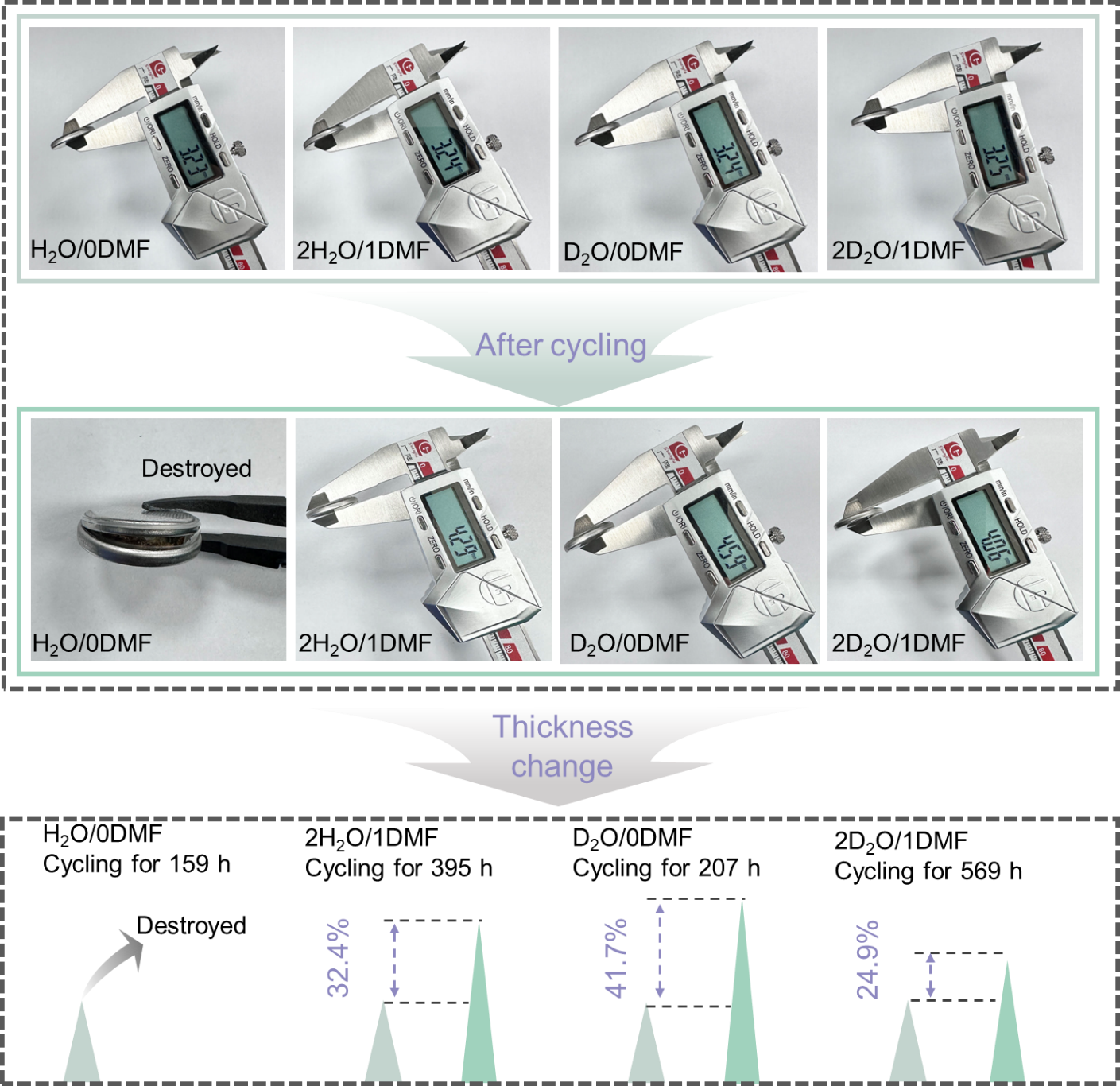


***Figure* *S24.*** The thickness change of battery with different electrolytes after cycling at 0.05 mA cm^-2^.


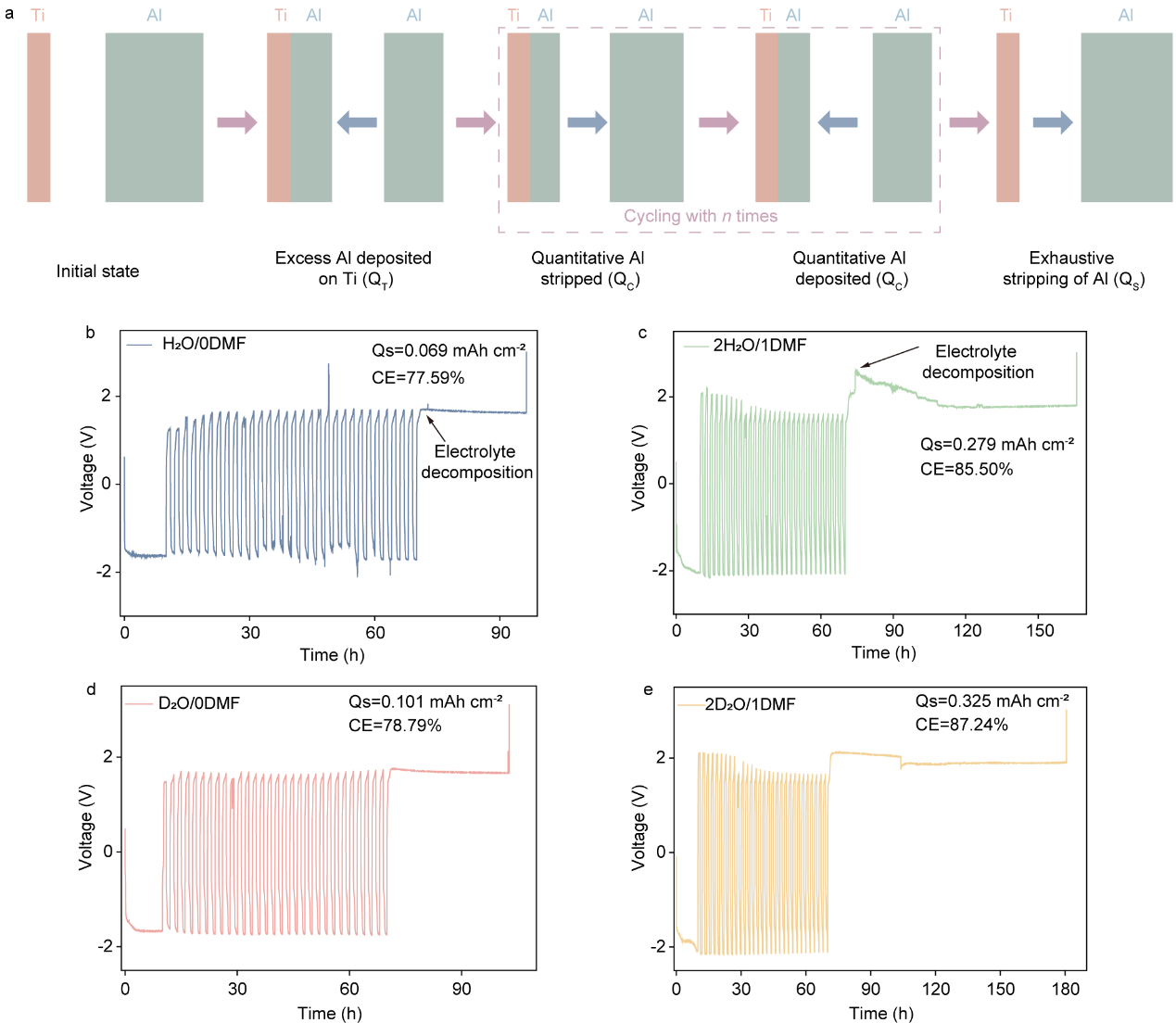


***Figure* *S25.*** CE analysis. (a) Schematic diagram of cycling Al||Ti cells for CE measurement. The measured voltage versus time plot for 30 cycles in the electrolyte with (b) H_2_O/0DMF. (c) 2H_2_O/1DMF. (d) D_2_O/0DMF. (e) 2D_2_O/1DMF. In this electrochemical test, we initially deposited an Al layer with a capacity of 0.6635 mAh cm^-2^ (Q_T_) on a Ti substrate. Subsequently, we performed 30 stripping/plating cycles using a smaller Al portion (Q_C_ = 0.06635 mAh cm^-2^). Following these cycles, we conducted exhaustive stripping of the remaining Al reservoir until reaching the cut-off voltage. Notably, upon Al depletion, water splitting and corrosion reactions generated a distinct voltage plateau. The final stripping charge (Q_S_) was determined from the onset of voltage drop, marking the complete removal of the Al reservoir. And the average CE over 30 cycles is calculated as equation (1)^17^.

CE = (nQ_C_ + Q_S_)/( nQ_C_ + Q_T_) (1)


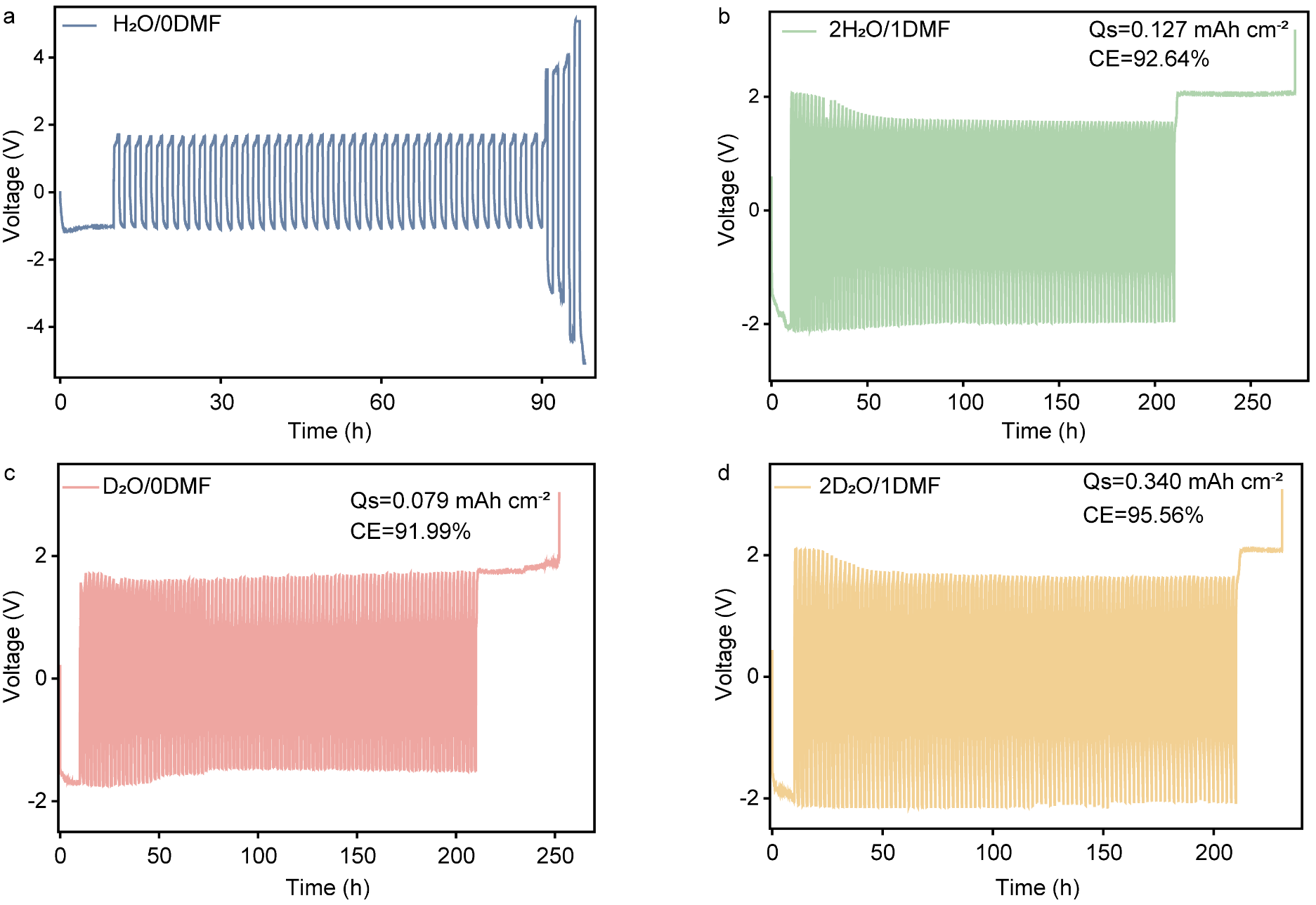


***Figure S26*.** The measured voltage versus time plot for 100 cycles in the electrolyte with (a) H_2_O/0DMF. (b) 2H_2_O/1DMF. (c) D_2_O/0DMF. (d) 2D_2_O/1DMF. Consistent with Supplementary Fig. S22-23, the terminal stripping phase exhibits characteristic voltage evolution. It shows an initial voltage rise correlating with aluminum stripping, followed by decline upon metallic Al depletion and subsequent electrolyte decomposition. Linear sweep voltammetry and cyclic voltammetry confirm water electrolysis dominance at a lower potential, establishing the extended voltage plateau as parasitic reaction signature. The potential inflection point marks the aluminum-stripping to side-reaction transition. Hence, the Qs was determined based on the point.


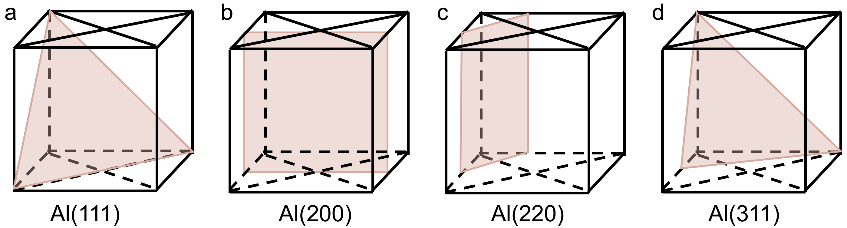


***Figure S27.*** Schematic diagram of the Al with a (111, 200, 220, 311) crystal plane.


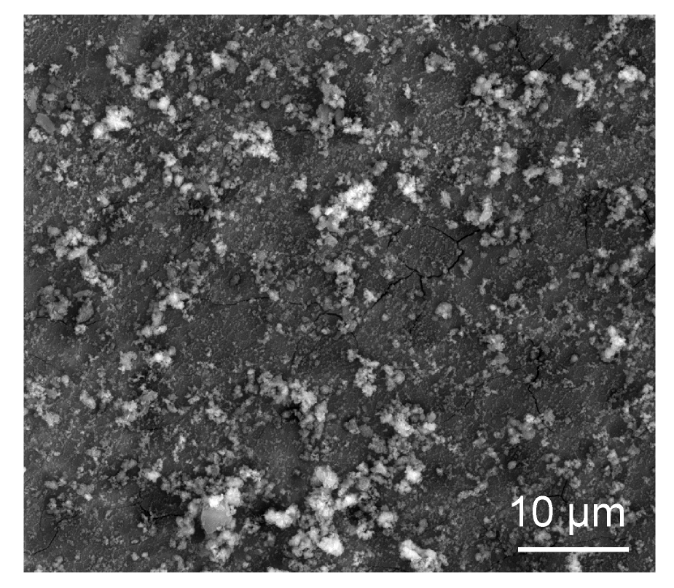


***Figure* *S28.*** SEM images of Al electrode before cycling.


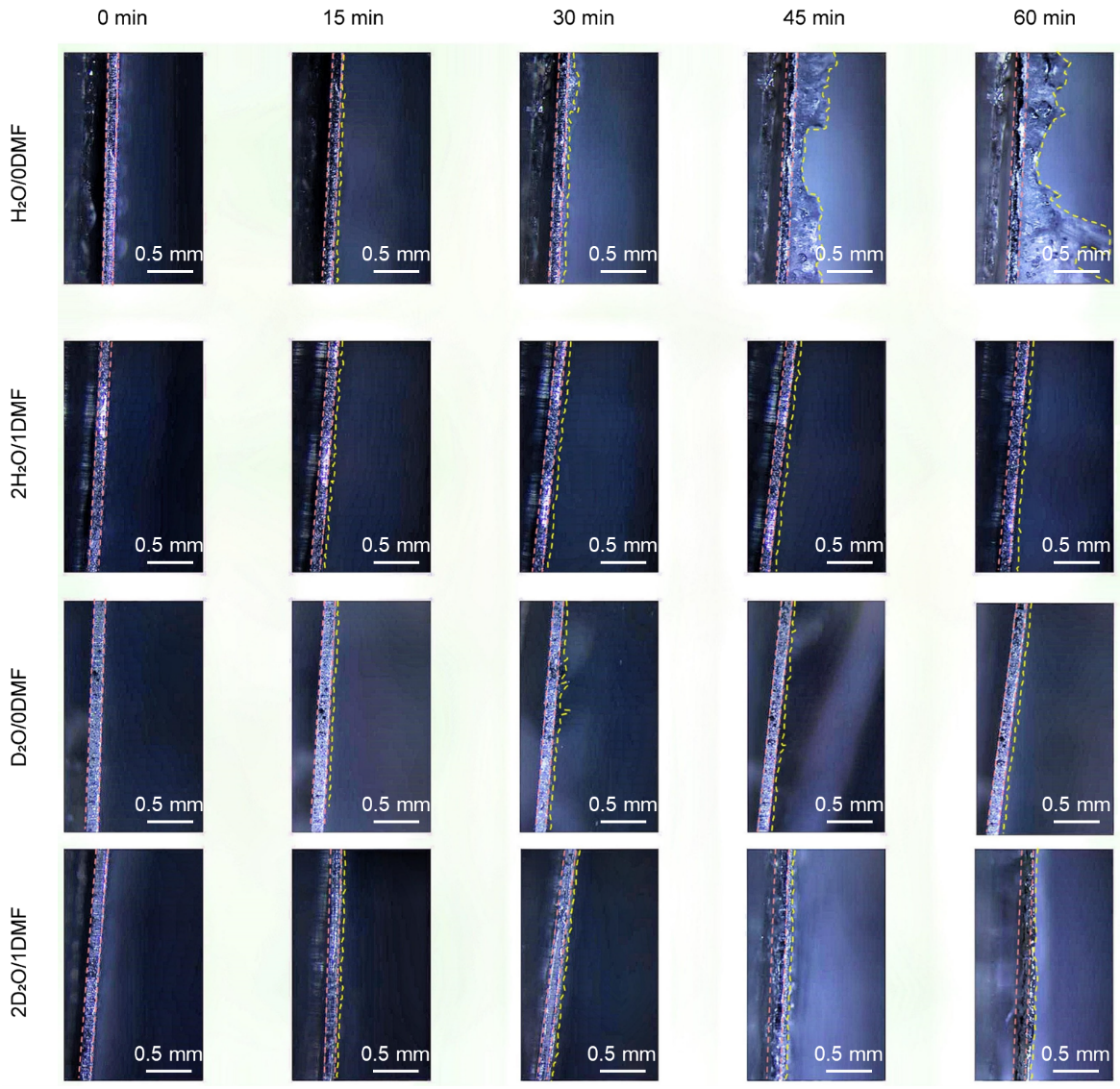


***Figure* *S29.*** In situ recording optical patterns of Al deposition on the anode side in Al||Al symmetric cell at 0.05 mA cm^-2^.


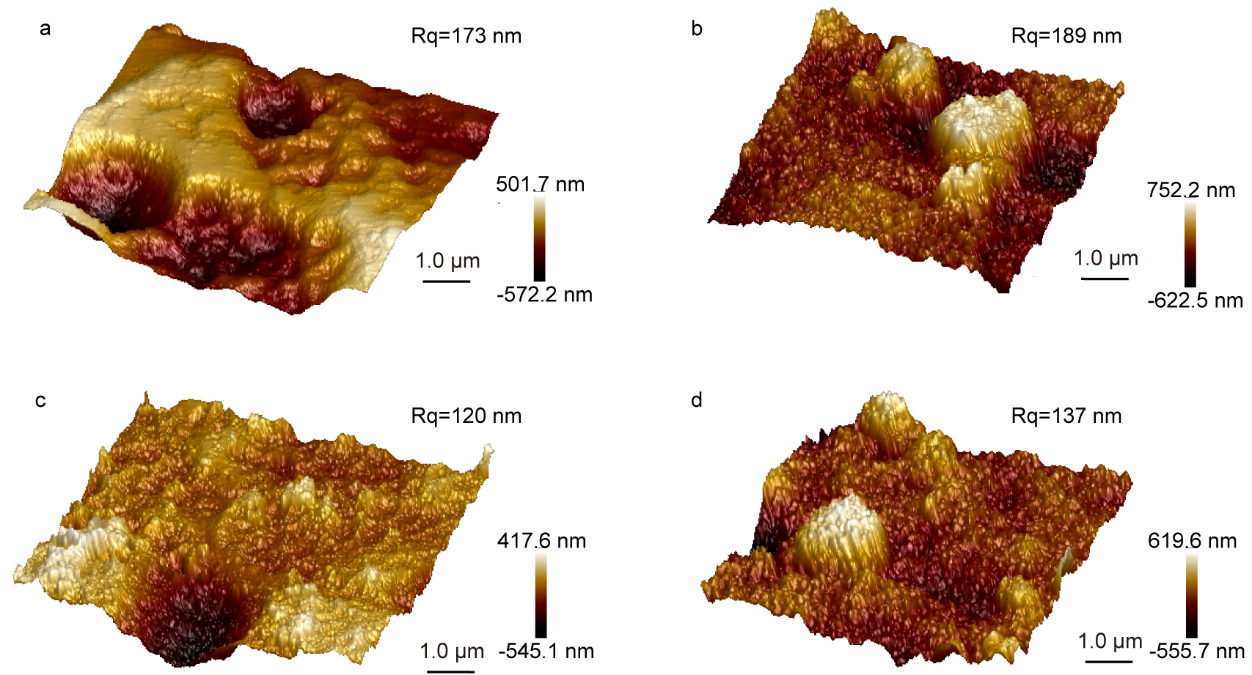


***Figure* *S30.*** 3D morphology for the Al electrode after cycling at 0.05 mAcm^-2^. (a) H_2_O/0DMF. (b) 2H_2_O/1DMF. (c) D_2_O/0DMF. (d) 2D_2_O/1DMF.

**Reference**

1 Frisch, M., Trucks, G. & Schlegel, H. Gaussian 09. Revision d. 01 [cp]. *J Wallingford CT: Gaussian Inc* (2013).

2 Stewart, J. J. P. et al. Mopac (22.0.4). *Zenodo* (2022).

3 Grimme, S., Antony, J., Ehrlich, S. & Krieg, H. A consistent and accurateab initioparametrization of density functional dispersion correction (dft-d) for the 94 elements h-pu. *J. Chem. Phys.* **132** (2010).

4 Weigend, F. & Ahlrichs, R. Balanced basis sets of split valence, triple zeta valence and quadruple zeta valence quality for h to rn: Design and assessment of accuracy. *Physical Chemistry Chemical Physics* **7** (2005).

5 Lu, T. & Chen, F. Multiwfn: A multifunctional wavefunction analyzer. *J. Comput. Chem.* **33**, 580-592 (2012).

6 Humphrey, W., Dalke, A. & Schulten, K. Vmd: Visual molecular dynamics. *J. Mol. Graphics* **14**, 33-38 (1996).

7 Szilárd, P., Abraham, M. J., Kutzner, C., Hess, B. & Lindahl, E. Tackling exascale software challenges in molecular dynamics simulations with gromacs. *Lecture Notes in Computer Science* **8759**, 3-27 (2015).

8 Martínez, L., Andrade, R., Birgin, E. G. & Martínez, J. M. Packmol: A package for building initial configurations for molecular dynamics simulations. *J. Comput. Chem.* **30**, 2157-2164 (2009).

9 Kaminski, G. A., Friesner, R. A., Tirado-Rives, J. & Jorgensen, W. L. Evaluation and reparametrization of the opls-aa force field for proteins via comparison with accurate quantum chemical calculations on peptides. *J. Phys. Chem. B* **105**, 6474-6487 (2001).

10 Linse, J.-B. & Hub, J. S. Three- and four-site models for heavy water: SPC/E-HW, TIP3P-HW, andTIP4P/2005-HW. J. Chem. Phys. 154, 194501 (2021).

11 Li, P., Song, L. F., & Merz Jr. K. M. Parameterization of highly charged metal ions using the 12-6-4 LJ-type nonbonded model in explicit water. J. Phys. Chem. B 119, 883-895, (2015)

12 Guo, J., Li, X. Z., Peng, J., Wang, E. G. & Jiang, Y. Atomic-scale investigation of nuclear quantum effects of surface water: Experiments and theory. *Prog. Surf. Sci.* **92**, 203-239 (2017).

13 McKenzie, R. H., Bekker, C., Athokpam, B. & Ramesh, S. G. Effect of quantum nuclear motion on hydrogen bonding. *J. Chem. Phys.* **140**, 174508 (2014).

14 Xu, X., Chen, Z. & Yang, Y. Molecular dynamics with constrained nuclear electronic orbital density functional theory: Accurate vibrational spectra from efficient incorporation of nuclear quantum effects. *J. Am. Chem. Soc.* **144**, 4039-4046 (2022).

15 Zhang, D., Zhang, Z., Jiang, W., Gao, Y. & Wang, Z. Effect of confinement on water rotation via quantum tunnelling. *Nanoscale* **10**, 18622-18626 (2018).

16 Horsewill, A. J. Quantum tunnelling in the hydrogen bond. *Prog. Nucl. Magn. Reson. Spectrosc.* **52**, 170-196 (2008).

17 Adams, B. D., Zheng, J., Ren, X., Xu, W. & Zhang, J. G. Accurate determination of coulombic efficiency for lithium metal anodes and lithium metal batteries. *Adv. Energy Mater.* **8**, 1702097 (2017).
